# How Domestic Demand Drives Coffee Export-Basket Upgrading: Pathways to Agri-Food System Transformation

**DOI:** 10.3390/foods15183240

**Published:** 2026-09-14

**Authors:** Ying Hu, Yongle Xie

**Affiliations:** School of Economics, Yunnan University, Kunming 650504, China

**Keywords:** coffee export-basket upgrading, domestic market absorption, demand maturity, resource orchestration, agri-food systems

## Abstract

The coffee industry in developing countries has long been locked into the lower end of the global value chain, and external governance mechanisms such as fair trade have struggled to fundamentally reverse this situation. This study adopts a mixed-methods research design, using the coffee industry in Yunnan, China, as a case study to reveal the micro-level mechanisms by which domestic demand drives industrial upgrading. Building on these case-derived mechanisms, it then uses panel data from 96 major coffee-producing and coffee-consuming economies from 2012 to 2024 to examine their macro-level implications, including national-level relationships, nonlinear patterns, and cross-country heterogeneity. The study shows that both factors generally drive export upgrading and exhibit a “promotion—inhibition—re-promotion” pattern characterized by a dual-threshold nonlinearity. The case study reveals that the inhibition phase stems from a mismatch between market expansion and demand structure upgrading, where homogeneous capacity growth temporarily offsets the effects of quality upgrading. Heterogeneity manifests in three distinct patterns: producing countries exhibit only a significant domestic market absorption effect; traditional consuming countries are primarily characterized by path dependence; and emerging consuming countries achieve dual-driven growth in both scale and quality. By bridging macroeconomic effects and microeconomic logic, this study clarifies the conditions under which domestic demand drives product-end upgrading along the value chain, providing a reference for different economies to formulate differentiated upgrading strategies.

## 1. Introduction

Within the global division of labor, developing countries have long faced a structural disadvantage characterized by insufficient capture of value added, and their lock-in at the lower end of global value chains has consistently been a major focus of research in development economics and international trade [1,2,3]. The coffee industry is a prime example of this asymmetry in the division of labor and distribution of value along the value chain. Coffee-producing countries primarily handle cultivation and primary processing, while value-added activities such as roasting, quality control, branding, and retail are largely concentrated in consumer markets. As a result, producers derive relatively limited profits from raw material transactions, while coffee products can achieve higher market value in downstream processing, branding, and retail. This phenomenon—in which limited returns on the production side coexist with expanding value on the consumption side, and product quality is disconnected from value capture—is known as the “coffee paradox” [4]. Consequently, some coffee-producing countries have long relied on the export of raw products (such as green coffee beans), while the transformation and upgrading of their industries have been constrained by value chains shaped by buyer-driven governance, international standards, and the division of labor. Given that external buyer-driven governance persists and is unlikely to change in the short term, an important question is whether coffee-producing countries can leverage their domestic markets to create an upgrading dynamic distinct from export-dependent growth. Specifically, this study examines how domestic market absorption contributes to coffee export-basket upgrading and how this relationship is conditioned by demand maturity and national market structure.

To address this question, we need to clarify the theoretical conditions and constraints of producer upgrading. Existing research indicates that the upgrading of producers cannot rely solely on integration into external global value chains, but must depend on the synergistic effects of value-added stages within the value chain and domestic market conditions. The market power of international buyers may drive down producer prices and reduce investment in quality, and stricter environmental standards may also be accompanied by a decline in value capture on the production side [5,6]. Whether external market access and the alignment of standards can translate into functional upgrades still depends on local institutional coordination, certification arrangements, and transactional relationships among value chain actors [7,8,9]. Therefore, to explain the upgrading of producers, it is also necessary to examine the value-added stages within the value chain and the organizational conditions that support them. At the broader agri-food-system level, food systems comprise the consumption, production, and processing of food products, while food markets play a critical role in connecting these activities with consumers. The ability of food products to reach consumers and gain market acceptance is influenced by affordability, availability, consumer needs, convenience, logistics, product type, quality, and quantity [10]. Moving from this system-level perspective to the agri-food value-chain level, processing and logistics are important sites of value addition and technology transfer, while information exchange, market organization, and supply-chain finance provide the foundation for the development of these sectors [11,12,13]. However, these value-chain capabilities and organizational supports do not automatically lead to upgrading; their effectiveness also depends on whether the market can absorb the additional output and send signals regarding quality improvements. Demand-side research indicates that the size of the domestic market can provide a foundation for the allocation of fixed costs and the accumulation of capabilities, while an upgrade in the structure of demand will guide enterprises to adjust their export product portfolios and shift toward quality-based competition [14,15,16]. This foundation of market size and quality indicators is also evident in the coffee industry. Quality information, such as flavor descriptions, influences consumers’ perceptions of products and their willingness to purchase [17], and the expansion of China’s coffee market has also provided demand-driven impetus for specialty coffee cultivation and supply chain optimization [18]. Overall, existing research has provided a relatively thorough examination of global value chain governance, the value-added stages within the agri-food value chain and their organizational conditions, as well as the role of domestic demand in industrial upgrading; however, it has not yet explained, within a single framework, how domestic market absorption leads to an upgrade in the coffee export basket through resource reallocation. At the same time, while the scale and quality of demand are typically treated as independent factors, there has been a lack of systematic research into whether the market absorption effect changes direction as demand maturity crosses different thresholds, and how this relationship manifests in the market structures of different countries.

To simultaneously identify the mechanisms underlying the domestic demand-driven upgrading of the coffee industry and the transnational constraints on this process, this study employs a mixed-methods research design combining “qualitative case study and quantitative empirical analysis.” Specifically, from a qualitative perspective, as domestic coffee consumption in China surges, the country is undergoing a historic transformation from a mere “raw material exporter” to a “hub for both production and consumption,” providing an ideal context for examining the relationship among evolving demand, resource reallocation, and value chain upgrading. Therefore, this study selects the Yunnan region—which accounts for more than 98 percent of China’s coffee production—as the subject of a qualitative single-case study. This study conducted semi-structured interviews with 362 representative entities (including companies, government agencies, and other relevant organizations) across the coffee industry chain. A total of 1628 individuals participated in the interviews, which served as the basis for the qualitative research data. Subsequently, based on the qualitative data, this study employs grounded theory to uncover the micro-level processes through which domestic demand influences product-side upgrading in the coffee industry, examining how industry actors respond to changes in demand and drive the upgrading of export product structures through resource reallocation and capacity building. Qualitative research has found that China’s coffee industry has evolved from a stage of piecing together resources during the raw-material production phase, through a stage of capacity restructuring and transitional friction during the shift toward specialty coffee, to a stage of supply chain and network resource coordination in the brand-building transformation phase. This phased evolution indicates that the role of the domestic market in facilitating export upgrading depends on the maturity of demand and the degree to which industrial capabilities align with the structure of demand, and may exhibit nonlinear changes. However, whether these case-derived implications are observable and the limits of their applicability across different national market structures still need to be examined through a broader body of cross-national evidence. Building on the findings of the case study, this study further constructs balanced panel data for 96 countries covering the period 2012–2024 to examine the relationship at the national level—and cross-country variations—between domestic market absorption, demand maturity, and the upgrading of the coffee export basket. It employs a system GMM model to estimate the overall relationship between domestic market absorption and demand maturity and the technical complexity of the coffee export basket, and examines their nonlinear characteristics and cross-country heterogeneity using a static panel threshold model and group-specific regression. Cross-country estimation results indicate that domestic market absorption and demand maturity are generally significantly correlated with the upgrading of the coffee export-basket; as demand maturity crosses two thresholds, the estimated coefficient for domestic market absorption exhibits a “promoting—inhibiting—re-promotion” pattern, which finding aligns with the phased mechanisms identified in the case study, such as sunk costs, technical barriers, and capacity restructuring. The results of the group analysis further show that the aforementioned relationship varies depending on a country’s market structure: coffee-producing countries primarily exhibit a market absorption effect, traditional coffee-consuming countries display a strong inertia toward upgrading, while emerging coffee-consuming countries exhibit both market absorption and the effects of demand maturity.

Compared with the existing literature, the marginal contributions of this study are primarily reflected in three aspects. First, theoretical contributions. This study introduces the theory of local market effects and the resource orchestration perspective into research on the upgrading of the export segment of the global coffee value chain. It constructs a cross-level explanatory framework comprising “domestic market absorption—resource reorganization among industry actors—increased technological complexity of the coffee export-basket,” and identifies demand maturity as a conditioning factor for the market absorption effect. Consequently, it expands the existing demand-side explanation—which posits the parallel roles of scale and quality—to a logical framework characterized by phased and nonlinear dynamics. Second, empirical contributions. Based on a case study in Yunnan, China, this study examines the context in which China functions both as a coffee-producing country and a rapidly growing emerging consumer market. It reveals the phased processes of resource reallocation among industry actors and export-side upgrading as domestic demand evolves. Building on this, the study utilizes cross-country panel data to further examine the observable manifestations of these mechanisms at the macro level. The study further reveals the nonlinear changes in market absorption effects as demand maturity crosses different thresholds, as well as variations in this relationship across countries with different market structures, providing evidence at both the micro and macro levels for understanding the conditions and limits under which domestic demand drives the upgrading of the export segment of the global coffee value chain. Third, methodological contributions. This study integrates a longitudinal process analysis based on grounded theory with transnational dynamic panel analysis and threshold identification within a single research framework, thereby providing a multilevel examination of industrial evolution, aggregate statistical relationships, and conditional boundaries. Compared with studies that rely on single case studies or estimates of average effects, this design broadens the scope of macro-level empirical analysis while preserving information on the context and processes of industrial evolution, thereby providing a hybrid research approach for the export-oriented upgrading of the agri-food industry that integrates both mechanistic explanations and transnational testing.

## 2. Theoretical Framework and Research Hypotheses

### 2.1. Domestic Market Absorption and Functional Upgrading

Domestic market absorption refers to the actual extent to which a country’s domestic market absorbs the output of its domestic industries relative to export markets, reflecting the relative importance of local demand in the realization of domestic output. Based on local market effects, under conditions of increasing returns to scale, greater domestic demand can expand the effective market available to firms, driving the concentration of production activities within the country and creating an export advantage [19,20]. Research at the firm level also indicates that domestic demand can enhance a firm’s position in the global value chain through economies of scale, economies of scope, and technological spillovers [14]. For industries that serve both domestic and international markets, local demand can only form a sales base that supports local investment and production organizations if it continues to absorb domestic output, thereby further transforming the advantages of market scale into production and processing capacity.

In the coffee industry, this effect is primarily transmitted through the allocation of fixed inputs in the post-harvest stage. Coffee cultivation is constrained by land and climate conditions, while post-harvest stages—such as processing, logistics, and wholesale—require greater capital, technology, and organizational resources, and are also critical links in the agricultural and food value chain for generating value-added [11,21]. Activities such as roasting, extraction, quality testing, and packaging all rely on specialized equipment, technical training, and investments in quality management. When domestic market demand is weak, locally produced processed coffee lacks stable sales channels, making it difficult for related facilities to maintain high capacity utilization rates. As a result, unit fixed costs rise and pressure to recoup investments increases, making it more likely for companies to continue relying primarily on green coffee bean exports. As the domestic market becomes more receptive to domestically produced goods, sustained local sales can improve equipment utilization, spread out the initial processing costs, and help companies build up their processing, quality control, and product development capabilities through repeated production. In terms of the structure of export products, research on the coffee trade classifies green coffee beans, roasted coffee, and instant coffee as product forms with varying degrees of processing, and finds that the level of industrialization and distance from processing centers are correlated with coffee value added [22]. The accumulation of processing capacity will ultimately be reflected in the shift in export products from primary forms to more highly processed forms.

In addition to the allocation of fixed investments, domestic market absorption may also be facilitated by the formation of processing capabilities through the clustering of supporting industries. As local production gains greater access to domestic markets, supporting sectors such as equipment supply, technical services, and logistics are more likely to concentrate locally, thereby reducing the costs of production, trade, and technology acquisition through input-output linkages, specialized division of labor, and knowledge diffusion [23,24]. The mutual reinforcement between the allocation of fixed investments and the clustering of supporting industries enables processing capacity to operate sustainably in producing countries and further serve international markets, driving the expansion of coffee exports from primary products—such as green beans—to products requiring higher levels of processing, thereby enhancing the technological complexity of the coffee export-basket. Based on the above analysis, this study proposes the following research hypothesis:

**H1.**  *Domestic market absorption positively promotes coffee export-basket upgrading, as reflected in the export-basket sophistication of a country’s coffee industry.*

### 2.2. Demand Maturity and Mechanisms for Inducing Innovation

Demand maturity refers to the conditions and extent to which the domestic market consistently identifies, accepts, and pays for differentiated products in terms of quality, functionality, origin, brand, and sustainability. Unlike market absorption, which reflects the extent to which domestic output is absorbed by the domestic market, demand maturity indicates whether the market can generate a relatively stable demand and payment base for differentiated products. Consumer quality preferences influence the quality structure of domestic production and export destinations; markets with strong demand for quality products are more conducive to the production and export of high-quality goods [25,26]. Compared to a simple expansion in the scale of demand, an upgrade in the structure of demand is more likely to prompt companies to increase the variety of high-quality products and shift toward quality-based competition [16]. This means that the maturity of demand primarily influences the product direction and innovation priorities of companies in utilizing their existing production capacity.

In the coffee market, demand for differentiation is primarily driven by attributes such as flavor, origin, processing methods, convenience, brand, and sustainability. Consumers are willing to pay a premium for labels such as “fair trade,” “organic,” and “direct trade” [27]. For example, although consumers in Honduras still have limited awareness of sustainability labels, they have already demonstrated a willingness to pay a premium [28]. Descriptions of coffee flavors also influence consumers’ perceptions of the product and their willingness to purchase it [17]. These studies indicate that differentiating characteristics can generate observable price and purchase signals, and the persistence and scope of these signals determine whether the domestic market can provide stable returns for the relevant products.

As consumer preferences for processing methods, product forms, and convenience continue to translate into purchases, the expected returns on processed products—such as roasted coffee, coffee extracts, and instant coffee—will increase accordingly, prompting companies to improve their roasting, extraction, formulation, and packaging processes; meanwhile, sustained focus on origin, quality, and sustainability will drive companies to enhance their quality control, traceability systems, and coordination of upstream procurement. Once experience in product development and quality management has been accumulated in the domestic market, it can be further extended to export markets, driving the shift in coffee exports from raw materials to product categories requiring higher levels of processing and thereby enhancing the technical complexity of the coffee export-basket. Based on this, this study proposes the second research hypothesis:

**H2.**  *Demand maturity positively promotes coffee export-basket upgrading, as reflected in the export-basket sophistication of coffee exports.*

### 2.3. Resource Orchestration and Friction in the Process of Export Upgrading

The absorption capacity and maturity of demand in the domestic market provide, respectively, a foundation for absorbing industrial upgrading output and conditions for differentiated demand. The former supports the allocation of fixed inputs and the development of processing capacity, while the latter reflects the market conditions that sustain the consumption of differentiated coffee products and generate stable returns. The theory of resource orchestration posits that a stock of resources can only develop the competitive capacity to adapt to market changes through structuring, combination, and utilization [29]. Therefore, whether domestic market absorption can translate into an upgrade of the export portfolio depends on whether industry players can realign their equipment, technology, supply relationships, and sales channels in accordance with market conditions as reflected by the maturity of demand. In the coffee value chain, this process also involves cross-stage collaboration among processors, brands, producer organizations, technical service providers, and growers.

When demand maturity is low—that is, when market purchasing power and the capacity to support differentiated products are limited—companies primarily focus on producing standardized, price-driven products. At this stage, expanding market absorption can increase the utilization rate of existing production capacity, spread out the costs of basic processing, and support the accumulation of initial capabilities. As market conditions improve, consumer demand for attributes such as quality, origin, processing methods, and sustainability is gradually increasing; however, the pricing basis and market returns for differentiated products have not yet stabilized. Existing equipment, technology, and supply relationships cannot directly meet the requirements of new products, so companies must bear the costs of resource reorganization, such as equipment upgrades, technology training, compliance with standards, and supply chain coordination. In addition to the specialized investments and sunk costs inherent in existing low-end production systems, this may also reinforce path dependence, allowing strong market absorption to continue to sustain the existing benefits of standardized production. Some studies indicate that financing conditions, technical support, and coordination among stakeholders influence the adaptability of different segments of the coffee value chain [30]; compliance costs resulting from process upgrades may also suppress exports in the short term, and the benefits of such upgrades will only gradually become apparent through the accumulation of knowledge [31]. Therefore, when differentiated demand has not yet established a stable market foundation, the costs of resource reallocation may offset the economies of scale resulting from market absorption.

As demand matures further and the market’s capacity to absorb differentiated coffee products and its consumer spending power stabilize, the expected returns for companies undertaking technical training, equipment upgrades, and supply chain adjustments will increase accordingly. At this point, the domestic market can serve as an outlet that not only provides a steady sales channel for processed products but also helps companies spread the costs of resource restructuring and product development, thereby further extending their processing, quality control, and product development capabilities into export markets. Accordingly, the effect of domestic market absorption on coffee export-basket upgrading may change as demand matures, following a nonlinear pattern that progresses from promotion to inhibition and then to re-promotion. Based on the above analysis, this study proposes research hypothesis:

**H3.**  *The impact of domestic market absorption on coffee export-basket upgrading is conditioned by demand maturity and exhibits a stage-contingent nonlinear pattern of “promotion—inhibition—re-promotion.”*

## 3. The Evolution and Domestic-Demand-Driven Upgrading of China’s Coffee Industry: Evidence from Yunnan

Yunnan is China’s most important coffee-producing region, accounting for more than 98 percent of the country’s total coffee output. For a long time, Yunnan’s coffee industry has relied primarily on international markets and multinational buyers, with industry activities concentrated on cultivation, primary processing, and the export of green coffee beans; in contrast, value-added processing, brand building, and end-market development have been relatively underdeveloped. This development model has driven the expansion of cultivation scale and the formation of a basic industrial chain, but it has also placed local producers in a relatively passive position when it comes to price formation, product development, and value capture. In recent years, China’s coffee consumption market has expanded rapidly, providing a crucial foundation of demand for the transformation of Yunnan’s coffee industry. According to the *2025 China Coffee Industry Development Report*, the Chinese coffee market is projected to reach 218.1 billion yuan in 2025, with the freshly ground coffee segment exceeding 188 billion yuan; coffee consumption is expected to surpass 400,000 metric tons, and the number of coffee shops is projected to reach 215,000 [32]. During the same period, the coffee cultivation area in Yunnan was approximately 1.463 million mu, with a green coffee bean output of 138,900 metric tons; high-quality coffee was exported to 29 countries and regions [33]. The expansion of the market, the diversification of consumption scenarios, and the growth of the sales network have amplified demand signals related to origin, quality, flavor, processing methods, and branding, driving the Yunnan coffee industry to gradually shift from raw material supply toward value-added processing, brand building, market expansion, and industrial integration.

Based on the aforementioned industrial evolution and changes in domestic demand, this study first employs a longitudinal single-case study approach, focusing on the coffee industry in Yunnan, to analyze its evolutionary process and the mechanisms driving its upgrading in response to domestic demand. Specifically, between May 2020 and December 2025, our research team conducted multiple field surveys in the five major coffee-producing regions of Pu’er, Baoshan, Dehong, Lincang, and Xishuangbanna. In total, respondents included coffee companies (E), service providers (A), research institutes (S), government agencies (G), and other relevant entities (O). In the descriptive sample, coffee enterprises, coffee farmers, government-agency representatives, and consumers accounted for 22.04%, 29.64%, 13.74%, and 34.58%, respectively, and their characteristics are reported in Supplementary Tables S4–S7. The interviews covered topics ranging from the industry’s development history, the responsibilities of key stakeholders, changes in the industry environment, corporate performance, cooperation along the industrial chain, and market interactions to cultivation, processing, distribution, brand building, and industrial integration (Table S3 in the Supplementary Materials). In addition to interview data, this study also draws on a combination of sources (I), including documents from government and industry organizations, corporate data, industry reports, and non-participatory observation (Table S8 in the Supplementary Materials). Section 3.1 will analyze the evolution of China’s coffee industry and its upgrading mechanisms based on this information.

### 3.1. Qualitative Case Design and Evolutionary Analysis of China’s Coffee Industry

#### 3.1.1. Evolutionary Stages of China’s Coffee Industry

To trace the evolution of China’s coffee industry, this study draws on historical data, industrial policies, and significant events to divide the industry’s development into four phases [34,35].

The industry inception and early Development Stage (1892–1987) was characterized primarily by the introduction of coffee cultivation and the initial emergence of industrial activities. At that time, the industry had not yet become deeply integrated into the global coffee value chain; export trade was sporadic and fragmented, and a large-scale product supply system had not yet been established. During the raw material source stage (1988–2013), as multinational buyers such as Nestlé entered the Chinese market and Yunnan launched large-scale, export-oriented cultivation, the industry was formally integrated into the global coffee division of labor. During this phase, the industry chain was highly concentrated in the cultivation segment and heavily dependent on orders from multinational buyers and external market demand. Exports consisted primarily of raw coffee beans and other primary raw materials, resulting in a significant loss of value added in the processing and branding segments. In the quality-oriented transformation stage (2014–2018), with the establishment of the Yunnan Coffee Exchange and the implementation of the specialty coffee auction mechanism, a trend emerged in China’s first-tier cities toward differentiated consumer demand for quality, origin, and traceability. Local enterprises gradually explored paths toward standardized production, refined processing, and product differentiation.Consequently, the structure of export products diversified, with the share of processed goods—such as roasted beans and instant coffee—steadily increasing. However, the sector as a whole remained dominated by the original equipment manufacturing (OEM) model, and exports under private labels remained limited in scale. Over the course of the brand-building transformation stage (2019–present), industrial policies have explicitly driven Yunnan’s coffee industry to transition from a “raw material base” to a “quality- and efficiency-oriented” model. As the domestic consumer market has expanded rapidly and demand has continued to mature, companies have begun to systematically integrate resources across various stages, including cultivation, processing, branding, sales, and supply chain coordination. In addition, the structure of export products has been further optimized. Some leading enterprises have launched their own branded export initiatives, and new export channels such as cross-border e-commerce have gradually emerged, forming an export landscape where raw materials, processed goods, and branded products coexist in a diversified manner.

**Figure 1:**
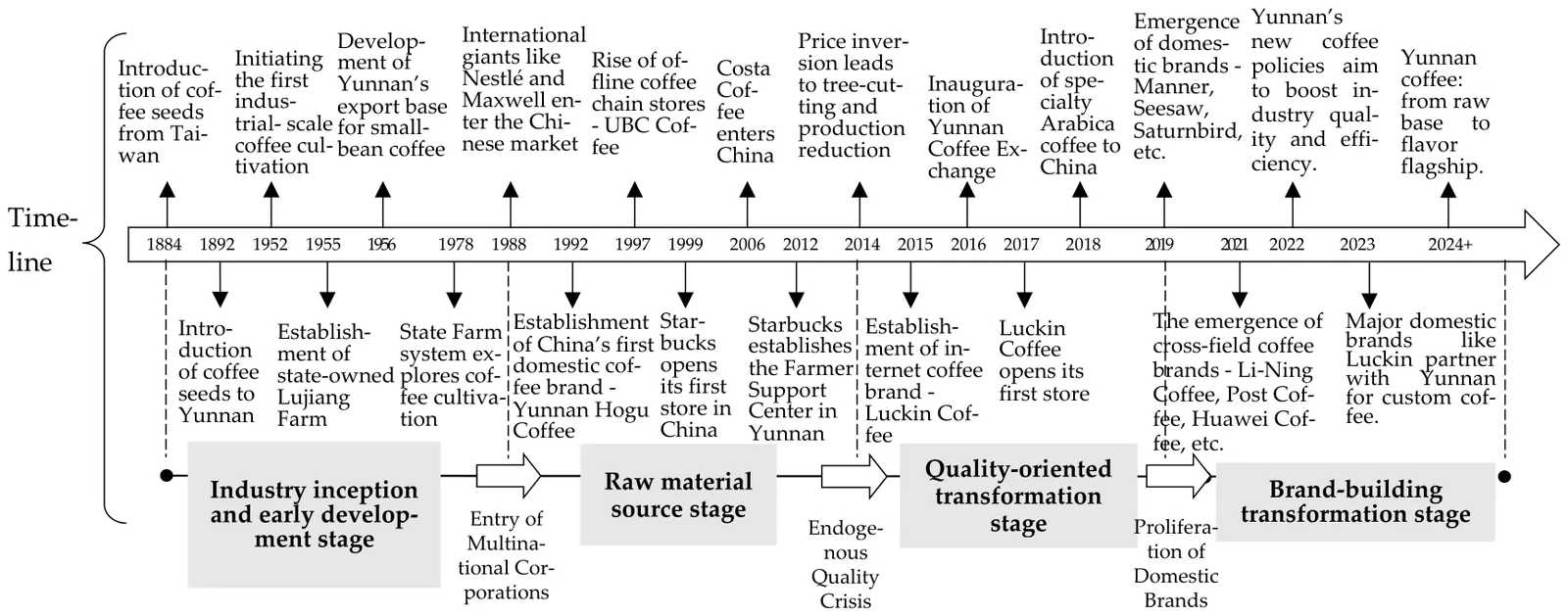
Timeline of the development of the coffee industry in China.

Given that, in its early stages, the Chinese coffee industry had not yet substantively integrated into the global division of labor network, subsequent theoretical analyses and discussions of mechanisms will focus on the final three core stages of evolution following the industry’s deep integration into globalization; representative events and milestones for each stage are shown in Figure 1.

#### 3.1.2. Three-Level Coding and Qualitative Evidence

(1) Level 1 coding: open-ended coding

Take the first stage of industrial development as an example (Table A1 of Appendix A), this study employs both line-by-line and event-by-event approaches to conduct open-coding of the interview data from this stage, with the aim of breaking down the data and identifying concepts that represent the original data segments. During the coding process, we focused on the industry roles, sources of resources, and development outcomes described by the respondents.

For example:

“In the early days, over 90% of our green coffee beans were exported directly in burlap sacks, primarily to fill the supply gap for bulk coffee beans in Europe and the United States.”

(Interviewee O11)

This passage not only indicates that coffee products are exported in the form of green beans, but also shows that the industry primarily serves as a supplier of low-value-added raw materials, with the value of its products and market potential constrained by bulk demand from Europe and the United States. Therefore, it is coded as “Reliance on bulk exports.”

Another example:

“Foreign-invested companies held absolute sway, setting purchase prices by deducting several dozen cents from New York futures prices, which drastically squeezed the net profit per mu that coffee farmers earned after a year of hard work.”

(Interviewee E21)

The core issue highlighted in this material is not simply “low prices,” but rather that multinational corporations control the power to acquire and set prices, while local producers lack bargaining power in the market. Therefore, it is coded as “Captured by multinational giants.”

An additional case:

“Yunnan’s greatest asset is its land located within the ‘golden latitude belt’ (Interviewee EX1); its unique geographical and climatic conditions provide the ideal environment for producing high-quality coffee.”

(Interviewee EX2)

This material transforms natural geographic conditions into coffee quality and competitive advantages for the industry, reflecting the proactive utilization of local resource endowments by industry stakeholders. Therefore, it is coded as “Leveraging local advantages.”

**Figure 2:**
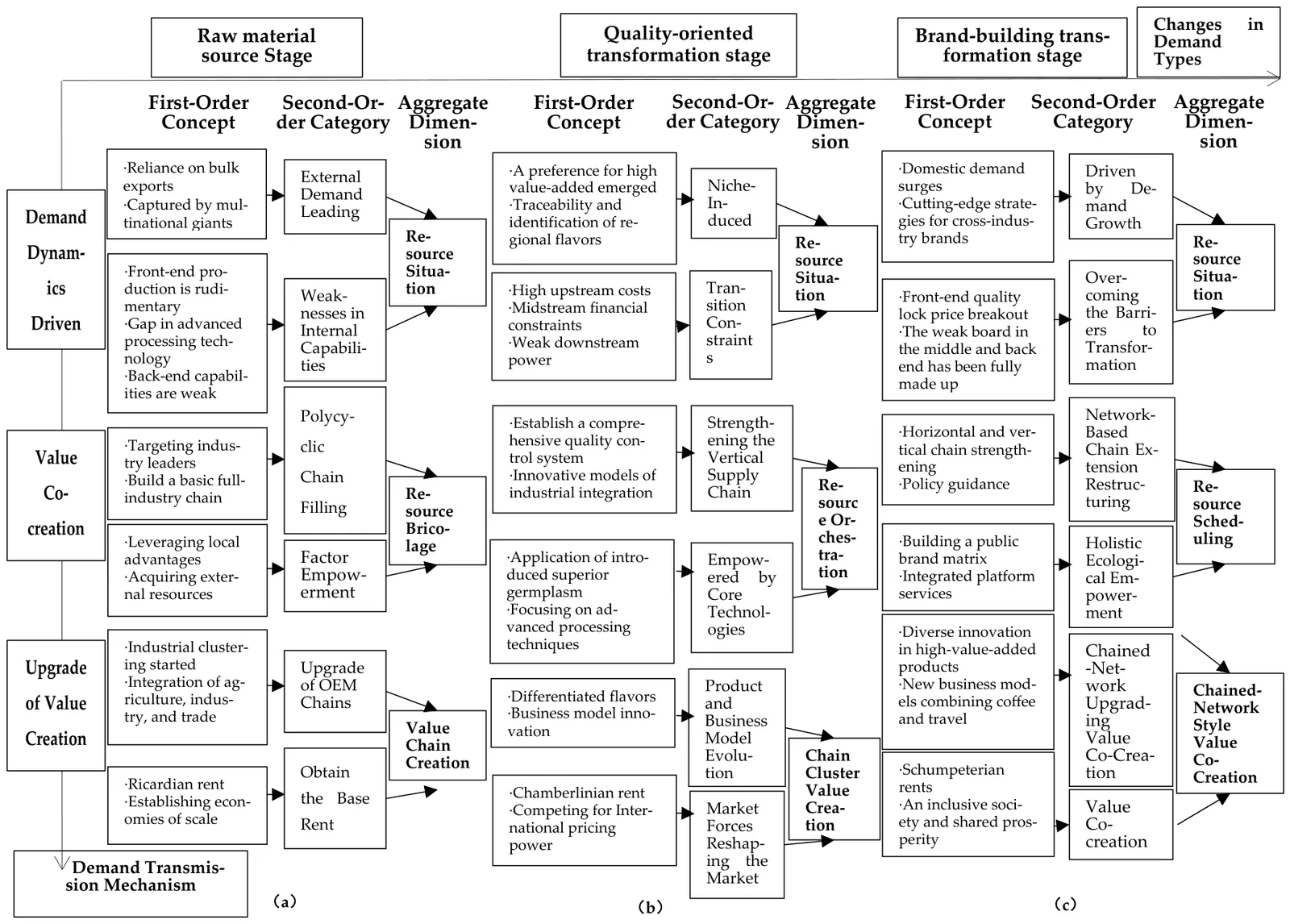
The evolution of China’s coffee industry. (**a**) Three-level coding framework for the “Raw material source stage”; (**b**) Three-level coding framework for the “Quality-oriented transformation stage”; (**c**) Three-level coding framework for the “Brand-building transformation stage”.

Following the same coding principles, this study further extracts the following themes from the interview data: “Acquiring external resources,” “Industrial clustering started,” “Integration of agriculture, industry, and trade,” “Front-end production is rudimentary,” “Gap in advanced processing technology,” “Back-end capabilities are weak,” “Targeting industry leaders,” “Build a basic full-industry chain,” “Ricardian rent,” and “Establishing economies of scale.” All of the above concepts directly correspond to the (a) listed in Figure 2.

(2) Second-level encoder: spindle-mounted encoder

Building on the first-level coding, this study classifies first-level concepts that share common connotations by comparing the subjects, resource relationships, capability requirements, and industrial roles associated with different concepts, thereby forming second-level categories.

“Reliance on bulk exports” and “Captured by multinational giants” reflect, respectively, the narrow focus of the export structure and the control exercised by foreign companies over market transactions. Together, these two factors indicate that industrial development at the raw material production stage is primarily driven by external market demand and the influence of multinational corporations, while local producers find themselves in a relatively passive position. Therefore, these two factors are grouped under the category of “External Demand Leading.”

“Front-end production is rudimentary,” “Gap in advanced processing technology,” and “Back-end capabilities are weak” refer to deficiencies in the cultivation, processing, and marketing stages, respectively. Although these first-level concepts refer to different stages of the industry, they all reflect relatively weak production capacity and value realization capabilities within the industry; therefore, they have been grouped under “Weaknesses in Internal Capabilities.”

“Targeting industry leaders” and “Building a basic full-industry chain” illustrate that the industrial entities improve their own development conditions by learning from industry leaders, perfecting processing procedures, and filling the gaps in the industrial chain. Together, these two reflect the process by which the industry reorganizes existing resources and industrial segments; therefore, they are grouped under the term “Polycyclic Chain Filling.”

Similar to the coding described above, the remaining first-level codes were grouped into “Factor Empowerment,” “Upgrade of OEM Chains,” and “Obtain the Base Rent,” resulting in a total of six distinct second-level categories.

(3) Level 3 coding: selective coding

Based on the secondary coding, this study further examines the functional differences in each secondary category in the process of industrial upgrading, and integrates the secondary categories with the same theoretical orientation into a higher-level aggregated dimension.

First, “External Demand Leading” and “Weaknesses in Internal Capabilities” together describe the fundamental conditions for development at the raw material production stage. The former reflects the external market environment in which the industry operates and its passive position in the international division of labor, while the latter reflects the industry’s internal shortcomings in production, processing, and market capabilities. These two factors reflect the resource base underlying industrial development from the perspectives of external constraints and internal conditions, respectively; therefore, they are both classified under the aggregate dimension “Resource Situation.”

Second, given a fixed set of resources, industry players do not passively accept constraints but instead actively reorganize their resources. “Polycyclic Chain Filling” and “Factor Empowerment” represent two complementary paths to restructuring: the former focuses on filling gaps in the industrial chain through learning and imitation, while the latter focuses on activating latent production capacity through the integration of factors of production. Since both are essentially creative assemblages and reuses of existing resources, they are collectively referred to as “Resource Bricolage.”

Ultimately, the cumulative effects of resource restructuring manifest as a qualitative transformation in the structure of the industry. “Upgrade of OEM Chains” reflects systematic improvements in the industrial chain structure (industrial agglomeration and the integration of agriculture, industry, and commerce), while “Obtain the Base Rent” reflects substantial improvements in business performance and terms of trade. Together, these two elements represent the outcome of the value chain’s transition from raw material supply to value creation; therefore, they are classified under “Value Chain Creation.”

To ensure the reliability and validity of the qualitative data analysis, this study relied on multiple corroborating data sources, reduced individual subjectivity through team discussions, and established a feedback mechanism to seek input from the original interviewees on preliminary coding conclusions in order to correct any biases. To test inter-coder reliability, two independent researchers who had not participated in the initial coding conducted blind, independent coding of a subset of the interview transcripts. The resulting Cohen’s kappa coefficient was 0.81, which is higher than the generally accepted threshold of 0.70, indicating that the coding framework exhibits high internal consistency. Regarding the few discrepancies that arose during the coding process, the team reached a consensus after discussion and consultation. Furthermore, we used the reserved 20% of the interview data to test for theoretical saturation; no new categories or theoretical dimensions emerged, confirming that the qualitative data had reached theoretical saturation. Based on the integration and analysis of the research findings, this study identified industrial upgrading pathways in the context of low-end lock-in in global value chains and iteratively refined these pathways through repeated comparison with existing literature until robust research conclusions were reached [36]. For details on the coding process, evidence from each stage, and excerpts from the interviews, see Appendix A and Supplementary Materials.

### 3.2. Domestic-Demand-Driven Upgrading Mechanisms of China’s Coffee Industry

#### 3.2.1. Raw Material Sourcing Stage: Entry into the Low-End Market and Initial Scaling Driven by Foreign Demand

During the raw material source stage (1988–2013), the evolution of China’s coffee industry exhibited typical characteristics of “exogenous passive embedding.” The domestic market was extremely limited, and the impetus for industrial upgrading relied almost entirely on the expansion of external demand driven by multinational buyers (such as Nestlé and Maxwell). Under this export-led mechanism, the effects of market expansion gradually took hold. Leveraging its unique geographical advantages and low-cost labor resources, Yunnan Province was able to gradually bridge the capability gaps across the industry’s upstream, midstream, and downstream segments. Leveraging the technology and knowledge spillover effects from multinational corporations, the province first integrated itself into the raw material supply segment of the global coffee division of labor at the cultivation stage. The industrial realities of this stage form the theoretical starting point of this study: without the buffer and support of local market economies of scale, local micro-entities are unable to bear the massive, fixed costs associated with upstream R&D and downstream brand building. Faced with “captive governance” dominated by multinational giants, local enterprises could only adopt a “Resource Bricolage” strategy. Through actions such as multi-loop chain supplementation and factor empowerment, although Yunnan’s coffee industry has expanded its cultivation scale and built up its primary processing capacity, its exports still consist mainly of raw products such as green beans, and the level of processing and product value-added remains relatively limited. Although these resource-patching efforts during this phase helped drive the industry’s transition from a “small, weak, and fragmented” state toward cluster-based cultivation, the complete loss of pricing power has, overall, resulted in a low-end cycle reliant on mere production expansion to sustain meager profits. Therefore, the upgrades in the raw-material production phase are primarily reflected in the expansion of basic production capacity and the completion of primary industrial chains, rather than in substantial improvements in processing depth, product structure, or the technical complexity of exports. For the coding categories, typical interview excerpts, and supporting evidence from the raw-material production phase, see Table A1 of Appendix A.

#### 3.2.2. The Transition to Premiumization: Structural Upgrades Driven by Niche Demand and the Growing Pains of Transformation

In the quality-oriented transformation stage (2014–2018), rising purchasing power and the emergence of niche demand for high-quality and traceable coffee strengthened quality-oriented market signals in major urban markets. The improving demand for quality forces local firms to try to exit the homogeneous price war and shift to refined management and product differentiation. However, this transition from “quantity” to “quality” encounters strong double threshold constraints and transition frictions. The structural lock-in effects accumulated because of the integration of the low-end raw material supply system in the previous phase have proven to be more persistent than expected. Since international buyers (such as Nestle and Starbucks) have long held pricing power, procurement standards are set unilaterally by them, and procurement prices are constantly constrained by international coffee futures prices. Furthermore, outdated primary processing facilities and equipment, coupled with a lack of standardized cultivation practices, have led to inconsistent coffee bean quality; this inherent quality crisis has further eroded the foundation needed for the local industry to make a leap forward. The high sunk costs accumulated from early integration into the low-end raw material supply system have created the first major obstacle to corporate transformation or exit. The capital and knowledge barriers to moving toward greater processing depth and a product mix with higher value-added are equally daunting. Massive capital investment in equipment is required by the meticulous processing of fresh fruit at the front end, while the mid-stream stage of deep processing faces even higher capital and technical barriers. During this transitional phase, when consumers’ willingness to pay for domestic brands has not yet fully matured, blindly investing in costly transformation efforts has instead triggered a period of internal competition—the industry has been caught in a tug-of-war between “catering to high-end demand” and “being constrained by low-end production capacity,” even falling into a “lemons market” dilemma at one point. Nevertheless, driven by the optimization of increasing demand maturity, local enterprises began to attempt to break through the industrial barriers established by multinational corporations through resource-allocation strategies such as vertical standardization and technological empowerment. These efforts enabled firms to develop differentiated products and establish an initial presence in quality-oriented market segments, thereby building momentum for future improvements in product quality, the accumulation of processing capabilities, and the upgrading of the export product mix. The coding categories, representative interview excerpts, and supporting evidence for the “transition to high-end products” phase are presented in Table S1 of Supplementary Materials.

#### 3.2.3. The Brand-Building Transformation Phase: Synergistic Upgrades and Value Co-Creation Driven by Evolving Market Demands

During the brand-building transformation phase (2019–present), the industry has experienced a comprehensive leap forward, fully demonstrating the synergistic effects of local market scale and increasing demand maturity. As China has emerged as the world’s fastest-growing coffee market, the massive domestic consumer base has provided local processing enterprises with a vast “pool for amortizing fixed costs.” This unprecedented benefit of local market scale has enabled enterprises to overcome the capital bottleneck in mid-tier deep processing and introduce state-of-the-art intelligent roasting and extraction production lines. At the same time, consumer preferences on the demand side have rapidly shifted from instant coffee to high-value-added end products such as freshly ground coffee, and market demand has transitioned from low-priced commercial beans to high-priced specialty coffee beans. A growing segment of consumers has shown interest in quality, origin, convenience, and brand attributes. The fragmentation of the demand side—marked by the explosive growth of multiple consumption scenarios such as freshly ground coffee, portable freeze-dried coffee, and ready-to-drink beverages—has acted as the “conductor” of resource allocation among micro-entities. By generating an innovation effect driven by demand, it has spurred process innovation, technological innovation, and category innovation, leading to the rapid emergence of domestic coffee brands (such as Luckin and Saturnbird coffee). Resource synergy through industrial chain extension is achieved through brand empowerment and network-based approaches, supporting the underdeveloped segments of the front-end value chain and driving coordinated upgrades in production and processing. Companies increasingly coordinate resources across cultivation, processing, branding, and distribution, while quality assessment and differentiated procurement begin to influence the organization of the chain. As domestic consumption volume and spending continue to expand, pricing has gradually shifted from being controlled by international buyers through international futures price premiums and discounts to being determined by local market supply and demand, and the introduction of international coffee cupping standards (such as COE and SCAA) has provided a relatively fair price discovery mechanism, incentivizing growers and processors on the supply side to spontaneously improve their cultivation, harvesting, and processing techniques to produce high-quality specialty coffee beans with irreplaceable flavors. This shift in pricing logic has profoundly altered the behavioral constraints of industry participants, gradually eroding the old pricing logic that relied solely on international futures prices as a benchmark, and has driven the formation of a new market mechanism based on local supply and demand, quality assessments, and brand premiums. At the same time, the “second-generation coffee farmers born in the 1990s” are gradually taking over their parents’ farming operations. By combining innovative brand marketing strategies with rapidly expanding e-commerce platforms, they have significantly broadened the distribution channels for specialty coffee and enhanced their brand value. Meanwhile, local governments have introduced a series of brand support policies—including measures to support coffee estate development and the certification of products with geographical indications—providing institutional safeguards for the transition to specialty farming and brand-driven transformation. Local actors have not only significantly reduced their dependence on multinational buyers and external end markets, but have also gradually generated innovation-driven returns centered on product innovation, brand premiums, quality control, and new business models, thereby achieving the co-creation of economic, social, and ecological benefits. The coding categories, representative interview excerpts, and supporting evidence for the brand-building transition phase are presented in Table S2 of the Supplementary Materials.

### 3.3. Case Findings

As can be seen from the above examples, the upgrading of China’s coffee industry has evolved from being “driven by external demand” to “upgrading of the demand structure,” and then to being “jointly driven by the size of the domestic market and the maturity of demand.” During the raw material source stage, the domestic market had not yet reached a significant scale, and the industry relied primarily on multinational corporations and international markets. By piecing together resources, the industry expanded its cultivation scale and improved its primary processing capabilities; however, overall, it remained confined to the low-value-added raw material supply segment. During the quality-oriented transformation stage, specific demands such as quality, origin, and traceability have gradually emerged. Industry players are driving product differentiation through supply chain coordination and technological empowerment, while simultaneously facing transition-related challenges such as sunk costs, technological investments, and insufficient market development. During the brand-building transformation stage, as the domestic market’s absorption capacity and demand maturity continue to increase, industry players have begun to achieve networked value co-creation through supply chain extension, public brand development, and platform-based services. The qualitative data mentioned above reflect the three-pronged logic of action addressed by the research hypothesis: First, the expansion of the domestic market provides a demand base that enables enterprises to expand production, spread fixed costs, and build processing capacity. Second, as consumer demand matures, consumer preferences—such as those regarding quality, origin, convenience, and brand—send signals indicating a focus on quality, driving product differentiation and deeper processing. Third, the shift from capability accumulation to transformation constraints to resource synergy across different stages reflects that demand maturity may have a conditional impact on the market absorption effect, causing it to exhibit a nonlinear pattern of “promotion—inhibition—re-promotion.”

In summary, this case study provides process-level evidence and micro-level explanations for the relationships proposed by H1 through H3 within the specific context of Yunnan, China. However, evidence from an individual country case alone is insufficient to determine whether these hypothetical relationships can also be observed at the “country-year” level in a broader national context, nor can it be used to estimate their overall association, threshold ranges, or cross-national differences. Therefore, this study further utilizes balanced panel data from 96 major coffee-producing and coffee-consuming countries covering the period from 2012 to 2024 to test H1–H3 at the macro level. Specifically, this study employs a system GMM approach to examine the overall relationship among domestic market absorption capacity, demand maturity, and the technical complexity of the coffee export basket; uses panel threshold regression to test whether the relationship between domestic market absorption capacity and the technical complexity of the coffee export basket exhibits nonlinear variations across different levels of demand maturity and employs subgroup regression to examine heterogeneity among countries under different market structures.

## 4. Cross-Country Evidence: Macro-Level Relationships and Boundary Conditions

Based on the overlapping coverage of multiple international databases, this study constructs a panel sample covering 96 countries from 2012 to 2024. In terms of the time frame, the period from 2012 to 2024 not only represents a continuous interval for which the relevant databases provide matching data but also aligns with the stages of industrial evolution identified in the case study: 2012–2013 marked the final phase of the raw material production stage; 2014–2018 corresponded to the transition to high-quality products; and 2019–2024 covered the main period of the transition to brand-building. Consequently, this time window provides a continuous, cross-national perspective for examining the phased changes between shifts in demand and the upgrading of the coffee export basket. In terms of cross-sectional coverage, the sample includes countries within the data range that have consistent records of coffee trade and consumption statistics, enabling it to reflect differences in coffee production, consumption, and trade activities across countries under a uniform framework. For individual missing values, where no alternative data was available, the moving average method was used to impute the values, ultimately resulting in balanced panel data.

### 4.1. Data Description

#### 4.1.1. Dependent Variable

This study uses coffee export-basket sophistication at the coffee-industry level to measure coffee export-basket upgrading, namely whether a country’s coffee exports are more concentrated in product categories typically exported by high-income economies. Drawing on the method for measuring export technological complexity proposed by Hausmann et al. [37], this study constructs a country-level measure of export-basket sophistication based on the export weights of six categories of coffee products and their revenue shares at the product level. In the context of the coffee industry, an increase in export-basket sophistication typically means that a country’s coffee export-basket is no longer focused solely on primary products such as green coffee beans but is shifting toward product categories with higher income content, such as roasted coffee, coffee extracts, and instant coffee. Because these product categories generally require additional processing and product development activities, the indicator provides indirect evidence of changes in processing depth. This makes EXPY suitable for capturing the product-side manifestation of upgrading in the global coffee value chain.

Step 1: Calculate the revenue share of coffee product k in year t.

(1) $${PRODY}_{kt}=\sum_{i}\frac{e_{ikt}/E_{it}}{\sum_{i}e_{ikt}/E_{it}}y_{it}$$

Step 2: Calculate the technological complexity of country i’s coffee industry exports in year t.

(2) $${EXPY}_{it}=\sum_{k}\frac{e_{ikt}}{E_{it}}{PRODY}_{kt}$$

where ${EXPY}_{it}$ is the technical complexity of coffee product export of country i in period t, ${PRODY}_{kt}$ is the technical complexity of coffee product k in period t, $e_{ikt}$ is the export value of coffee product k of country i in period t, $E_{it}$ is the total export value of coffee product of country i in period t, and $y_{it}$ is the per capita gross domestic product (GDP) of country i in period t. Export value data are sourced from the UNCOMTRADE database (with missing values supplemented by the CEPII database). Six coffee export products under the HS 6-digit classification (covering the main exports of the coffee industry) were selected and converted to real export values for the base year 2015 using the export value indices for each country and year, which are sourced from the WDI database. Per capita GDP for each country is sourced from the World Bank database, using constant U.S. dollars measured at 2021 purchasing power parity (PPP).

#### 4.1.2. Key Explanatory Variable

(1) Referring to the practice of Dai et al. [38], this study adopts the degree of market divergence (${DI}_{i}$) as the proxy variable of domestic market absorption (${DMS}_{it}$). The indicator captures the extent to which domestic consumption absorbs coffee-sector output relative to export dependence, and the calculation formula is as follows:
  (3) $${DI}_{i}\;=\;\frac{{Con}_{i\;}/\sum {Con}_{i}\;}{{Exp}_{i}/\sum {Exp}_{i}}$$
  where ${Con}_{i}$ and ${Exp}_{i}$ represent the domestic final consumption and exports of country i to an industry, $\sum {Con}_{i}$ and $\sum {Exp}_{i}$ represent the world’s domestic final consumption and exports to an industry, and ${DI}_{i}$ represents the degree of deviation between the consumption of the industry in country i and the export market. The larger the index is, the higher the proportion of local consumption in the industry, and thus the stronger the domestic market absorption capacity relative to export dependence. This study uses stock-adjusted apparent consumption to measure the domestic consumption of coffee in each country, which is obtained from the USDA, FAOSTAT, and UNCOMTRADE; the value of exports to the world is obtained from the UNCOMTRADE database.
(2) This study uses a country’s per capita GDP to proxy for its demand maturity. ${STRUC}_{it}$): This variable is used to characterize the purchasing power base that underpins differentiated coffee consumption. From the perspective of theoretical mapping logic, this macroeconomic indicator can, to a certain extent, reflect the evolving trends in consumer choices at the micro level. As per capita income rises, consumer preferences are no longer limited to low-priced, bulk products that merely satisfy basic drinking needs; instead, they are gradually shifting toward differentiated products characterized by specific origins, advanced processing, distinctive flavors, brand value, and sustainability, and consumers’ willingness to pay a premium is correspondingly increasing. Therefore, an increase in per capita GDP generally corresponds to a collective shift in consumer preferences toward high-value-added, differentiated products, and can be used to characterize the evolving structure of market demand. Rising income levels not only provide the purchasing power needed for demand diversification but may also drive supporting investments in areas such as processing, branding, and quality management, thereby encouraging companies to adjust their coffee export portfolios and upgrade toward products with higher value-added and greater processing complexity.

#### 4.1.3. Control Variable

In addition to the core explanatory variables of domestic market absorption and demand maturity studied in this study, there are other factors that affect the sophistication of the coffee export basket. This study considers the level of industrial development (proxied by the proportion of the manufacturing sector in GDP), natural resource endowment (proxied by the proportion of total natural resource rents in GDP), capital stock (proxied by the proportion of capital stock in GDP), the ratio of foreign direct investment to GDP and the exchange rate (the annual average of the official exchange rate of the local currency against the US dollar) as five control variables; the above indicators are sourced from the WDI database and the WGI database.

The measurement description and descriptive statistics of the variables are shown in Table 1. In order to coordinate the regression coefficients of each variable in the regression results, the data of each variable are adjusted by order of magnitude. Furthermore, as shown by the data in Table A2 and Table A3 in Appendix A, there are no serious multicollinearity issues among the core explanatory variables and control variables in this study, and the regression coefficients are not significantly distorted by linear dependencies among the variables.

### 4.2. Model Design

This study considers the fact that the sophistication of the coffee export basket exhibits a certain degree of inertia, meaning that the previous period’s coffee export mix influences the current period’s export mix. Therefore, the dependent variable is included in the equation, and its first-order lagged value is used as an instrumental variable [38]. Accordingly, this study specifies the following dynamic panel estimation equation:

(4) $${LnEXPY}_{it}\;=\;\beta_{0}\;+{\;\beta }_{1}{LnEXPY}_{i,\;t-1}\;+\;\beta_{2}Ln{DMS}_{it}\;+{\;\beta }_{3}{LnSTRUC}_{it}\;\;+\;\gamma Z_{it}\;+{\;\lambda }_{i}\;+{\;\eta }_{t}\;+\;\varepsilon_{it}$$

where $Z_{it}$ is a series of control variables, including ${Resource}_{it}$, ${Inflow}_{it}$, ${Manu}_{it}$, ${Gross}_{it}$, ${Lnofficial}_{it}$; $\lambda_{i}$ and $\eta_{t}$ are individual fixed effects and time fixed effects, respectively; $\varepsilon_{it\;}$ is the random disturbance term.

In addition, considering the possible nonlinear relationship between domestic market absorption and coffee export-basket upgrading, its promoting effect may be constrained by objective conditions such as demand maturity and natural resource endowment, which cannot be effectively identified by the general linear model. To this end, this study draws on the panel threshold model of Hansen [39] and, based on parameter estimation and hypothesis testing of the threshold value, deeply analyzes the threshold effect of the impact of domestic market absorption on coffee export-basket upgrading. Based on the benchmark model, this study takes demand maturity (${LnSTRUC}_{it}$) as threshold variables to construct the corresponding single threshold model:

(5) $${LnEXPY}_{it}\;=\;\alpha_{0}\;+\;\alpha_{1}Ln{DMS}_{it}\cdot I(K_{it}\;\leq \;\gamma )\;+\;\alpha_{2}Ln{DMS}_{it}\cdot I(K_{it}\;>\;\gamma )\;+\;\;\alpha_{3}K_{it}\;+{\;\alpha }_{4}Z_{it}\;+\;\lambda_{i}\;+{\;\varepsilon }_{it}$$

where I(·) is the indicator function, which is 1 when the conditions in parentheses are met, and 0 otherwise; $K_{it}$ is the corresponding threshold variable; γ is the threshold value to be estimated. After determining a single threshold, it is necessary to further test whether there are two or more thresholds.

(6) $${LnEXPY}_{it}={\;\alpha }_{0}+\alpha_{1}Ln{DMS}_{it}\cdot I(K_{it}\;\leq \;\gamma_{1})+{\;\alpha }_{2}Ln{DMS}_{it}\cdot I(\gamma_{1}\;<{\;K}_{it}\;\leq {\;\gamma }_{2})+\;\;\alpha_{3}Ln{DMS}_{it}\cdot I(K_{it}>\gamma_{2})+{\;\alpha }_{4}K_{it}+{\;\alpha }_{5}Z_{it}+{\;\lambda }_{i}+{\;\varepsilon }_{it}$$

Equation (6) is the two-threshold model, $\gamma_{1}$ and $\gamma_{2}$ are the first threshold value and the second threshold value, respectively, and the three-threshold model and so on until the test fails.

### 4.3. Results and Analysis of Empirical Testing

#### 4.3.1. Benchmark Regression Analysis

To examine the impact of domestic market absorption and demand maturity on coffee export-basket upgrading, and to effectively address potential issues of endogeneity and dynamic dependence, this study first verifies the stationarity of the variables using the LLC test and the Hadri unit root test, thereby ruling out the possibility of spurious regression. Building on this foundation, a dynamic panel model is constructed using the system GMM method.

Table 2 summarizes the estimation results of the dynamic panel benchmark regression. In the process of gradually incorporating the core explanatory variable and a series of control variables, this study employs the ‘collapse’ method to reduce the set of instrumental variables, thereby mitigating the problem of instrument proliferation [40]. The instrument-to-group ratios for the various models were 0.156, 0.167, and 0.219, respectively—all well below the threshold of 1—indicating that the number of instrumental variables relative to the number of cross-sectional groups falls within a reasonable range. Under these settings, the *p*-value for the AR(2) test is greater than 0.1, indicating that there is no second-order serial correlation in the model; the *p*-value for the Hansen over-identification test is also greater than 0.1, indicating that the instrumental variables generally satisfy the validity requirements and that there is no over-identification issue. The regression coefficients for the one-period lagged term of the dependent variable (L.LnEXPY) are all significantly positive at the 1% level (coefficients are 0.681, 0.635, and 0.592, respectively), indicating that coffee export-basket upgrading exhibits significant path dependence. Regarding the effect of domestic market absorption, the results in columns (1) through (3) of Table 2 show that the coefficient for domestic market absorption is significantly positive at the 1% significance level, strongly supporting the hypothesis H1 to be tested in this study. This suggests that countries with strong domestic market absorption capacity typically have a more diversified coffee export portfolio. Based on the case of Yunnan, China, domestic market expansion—by providing demand support for the accumulation of processing capacity and adjustments to the product mix—may serve as a key pathway through which the domestic market absorbs the impact of export upgrading, and provides a case study for understanding the positive relationship observed across multiple countries.

To further examine the association between demand maturity and export-basket upgrading, the estimation results in columns (2) and (3) of Table 2 indicate that the coefficient of the variable LnSTRUC, which represents demand maturity, is also significantly positive at the 1% significance level. Specifically, based on the regression results in Column (3), a 1% increase in demand maturity is associated with an approximately 0.082% increase in coffee export-basket sophistication, thereby confirming Hypothesis H2. The estimation results from the aforementioned cross-country sample indicate that the relationship between demand maturity and the upgrading of the coffee export basket, as revealed by the Yunnan case, also exhibits a corresponding macro-level correlation in a broader sample of countries. The pathways identified in the Yunnan case—such as demand-driven innovation, the accumulation of processing capabilities, and value chain synergy—can provide micro-level mechanisms to explain this positive correlation at the transnational level. Existing literature has also provided systematic theoretical foundations and empirical evidence for certain mechanisms from various perspectives, including innovation driven by quality demands, industrial upgrading spurred by market size, and technology diffusion within global value chains. Existing literature has also provided systematic theoretical foundations and empirical evidence for certain mechanisms from various perspectives, including innovation driven by quality demands, industrial upgrading spurred by market size, and technology diffusion within global value chains [25,41,42].

Taken together, the benchmark regression results indicate that both domestic market absorption and demand maturity contribute to improving the income content and complexity of the coffee export basket, thereby supporting H1 and H2.

#### 4.3.2. Threshold Regression Results

This study employs the bootstrap method and Stata 18.0 software to calculate the F-statistic by simulating the likelihood ratio through 500 rounds of bootstrapping and uses the corresponding *p*-values to test for the existence and validity of the threshold effect. The threshold regression results in Table 3 indicate that the association between domestic market absorption and coffee export-basket upgrading is not linear. Instead, it is constrained by a dual threshold imposed by demand maturity, exhibiting a nonlinear evolutionary pattern of “promotion—inhibition—re-promotion.” This provides conditional evidence consistent with the “deep transformation growing pains” hypothesis (H3) proposed in this study.

**Figure 3:**
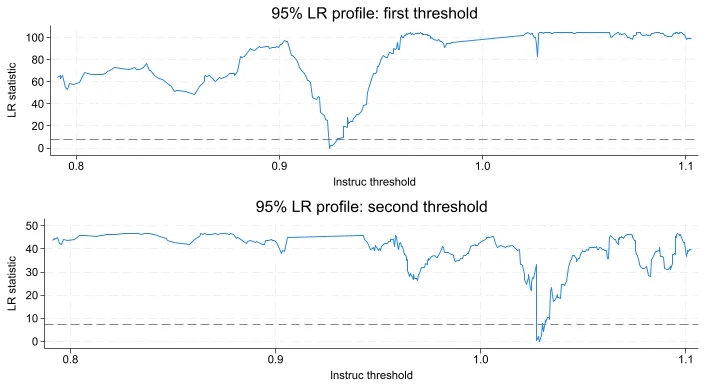
Likelihood-ratio profiles for the double threshold.

Specifically, the estimated value of the first threshold in Figure 3 is 0.9247, with a 95% confidence interval of [0.9239, 0.9251]. When demand maturity is low—that is, when LnSTRUC ≤ 0.9247—domestic market demand has a significant positive impact on the upgrading of the coffee export-basket, with a regression coefficient of 0.046 that is significant at the 1% level (Table 4). During this “initial expansion” phase, the growth in domestic basic demand may provide a market foundation for production expansion, improvements in primary processing capacity, and initial enhancements to the export product mix, thereby supporting enterprises in accumulating production experience and capital and driving an initial upgrade of the coffee export-basket. Once the demand maturity index crosses the first threshold (0.9247 < LnSTRUC ≤ 1.0307), the estimated coefficient for domestic market demand drops to −0.029 and is significant at the 1% level, indicating that a significant negative relationship has emerged between domestic market absorption capacity and the upgrading of the coffee export-basket. This phase may correspond to a period of adjustment during which the industry transitions from primary processing to products with higher value-added and greater complexity. Path dependence resulting from existing low-value-added production structures, combined with the capital investment in equipment, technical standards, financing conditions, and brand-building costs required for value-added processing, may make it difficult for the expansion of domestic demand to translate into a driving force for upgrading the export product structure in the short term. The estimated value for the second threshold is 1.0307 (Figure 3), with a 95% confidence interval of [1.0287, 1.0315]. When the demand maturity crosses the second threshold, namely LnSTRUC > 1.0307, the estimated coefficient of the domestic market demand rises to 0.030 and is significantly positive at the level of 1%, indicating that with the further improvement of the demand maturity, the absorptive capacity of the domestic market is again transformed into a positive force to promote the upgrading of the coffee export-basket.

### 4.4. Results of the Stability Test

To verify the robustness of the conclusions in this study, the following four methods are used to conduct the robustness test: ① Dealing with the endogeneity problem. There may be reverse causality between domestic market absorption of coffee and coffee export-basket upgrading; that is, stronger domestic market absorption may promote product-side upgrading of the coffee export-basket, while a more sophisticated coffee export-basket may also enhance domestic firms’ capabilities, brand reputation, and market expansion potential, resulting in potential endogeneity problems. Therefore, this subsection controls for the domestic market absorption effect as an endogenous variable and re-estimates the benchmark regression. The result is shown in columns (1)–(3) of Table 5. ② Changing the sample interval. Considering that the outbreak of COVID-19 in 2020 caused a severe impact on the global macroeconomy and logistics, the sample of that year may have abnormal fluctuations. To eliminate the interference of this extreme event on the estimation results, this study eliminates the single-year sample of 2020 and then estimates the model. The result is shown in column (4) of Table 5. ③ Replacing explanatory variables. This study measures the domestic consumption of coffee in each country based on the income of the coffee market in each country according to the Statista database and recalculates an alternative measure of domestic coffee demand in each country. In addition, to avoid a spurious correlation between the core explanatory variable and the dependent variable due to shared export data, we replaced it with the logarithm of per capita coffee consumption per 100 people in each country. The results are shown in columns (5) and (6) of Table 5, respectively. ④ Replacing the dependent variable. Given that export-basket sophistication primarily reflects the income content and product composition of the coffee export-basket and does not fully capture differences in quality and unit value within the same product, this study further employs export-basket sophistication adjusted for unit value as a variable for robustness testing.

The unit-value-adjusted indicator is calculated as follows:

(7) $${EXPY}_{it}^{UV}=\sum_{k}\frac{e_{ikt}}{E_{it}}{PRODY}_{kt}\frac{P_{ikt}}{P_{kt}}$$

Here, $P_{ikt}$ represents the unit value of coffee product k exported by country i in period t, and $P_{kt}$ represents the world average unit value of coffee product k in period t. If we substitute the export technical complexity adjusted for unit value, and the directions and significance of the core variables remain consistent, this indicates that the conclusions of this study still hold (Column (7) of Table 5) even after accounting for differences in unit value within the same product. In addition, this study further uses the share of exports of highly processed products as a supplementary robustness test to examine whether coffee exports have shifted from primary products, such as green coffee beans, to highly processed products, such as roasted coffee, coffee extracts, and instant coffee. This supplementary indicator provides a more direct measure of the processing-composition dimension than EXPY, although it does not measure the quality of individual products.

The formula is constructed as follows:

(8) $$E_{ikt}^{GBE}\;={\;E}_{ikt}^{Nominal}\;\times {\;CF}_{k}$$

(9) $${Upgrade}_{it}=\frac{\sum_{k\in DeepProcessed\;}E_{ikt}^{GBE}}{\sum_{k}E_{ikt}^{GBE}}$$

Here, ${CF}_{k}$ represents the conversion factor for different products: green coffee beans = 1.00, roasted coffee beans = 1.19, soluble coffee = 2.60 [43], $E_{ikt}^{GBE}$ represents the export value in green coffee bean equivalents, $E_{ikt}^{Nominal}$ represents the nominal export value of different products, and Upgradeit represents the proportion of processed coffee products in total exports, with a range of [0, 1]. The closer the value is to 1, the higher the proportion of processed products in the country’s coffee exports, and the stronger the evidence that the country’s coffee export-basket is shifting from raw-material products toward processed product categories. This supplementary indicator further captures the processing-structure dimension of product-side upgrading in the global coffee value chain. A value closer to 0 indicates that the country primarily exports primary products, such as green beans, and is situated at the raw material supply stage at the lower end of the value chain. In Table 5, Column (8) reports the regression results for the dependent variable adjusted by the conversion coefficient, while Column (9) reports the unadjusted results.

The results in Table 5 show that the conclusions of this study have good stability. Under the four robustness settings, the signs and significance of core variables do not change substantially.

### 4.5. Heterogeneity Analysis

Given that the drivers of coffee export-basket upgrading vary significantly across different countries within the coffee industry chain—with traditional consumer markets primarily relying on long-established processing, branding, and distribution capabilities, coffee-producing countries being more constrained by raw material supply and insufficient local effective demand, and some non-traditional markets exhibiting characteristics where consumption expansion drives adjustments in industrial capacity—estimates based on the entire sample may obscure these structural heterogeneities. This study defines emerging coffee-consuming countries as those that have already developed a certain scale of consumption and a foundation of purchasing power but have not yet entered the ranks of traditional mature consumer markets, and classifies them according to the sequence of “traditional market status—production role—local demand conditions.” First, based on the International Coffee Organization’s classification of traditional and non-traditional coffee markets [44], and taking into account the market roles established by the sample countries as of 2012, 24 countries—including the United States and Japan—were classified as traditional coffee-consuming countries. Second, among the remaining countries, the production role was identified based on whether their green coffee bean output in 2012 was greater than zero; countries that did not play a production role—whose coffee industry activities relied primarily on imports and domestic consumption—were classified as emerging coffee-consuming countries. For countries that are coffee producers, the following criteria are used to determine whether their domestic markets have the necessary consumption scale and purchasing power: domestic coffee consumption of at least 500,000 bags (60 kg per bag) and a per capita GDP of at least $4234.18.Countries that meet both criteria are classified as emerging coffee-consuming countries, such as China and Brazil; countries that do not meet both criteria are classified as coffee-producing countries, such as Ethiopia and Honduras. See Table A4 of Appendix A for the specific classifications.

As shown in Table 6, the coefficient for the domestic market absorption effect in coffee-producing countries is 0.033, which is significantly positive at the 1% significance level, while the coefficient for demand maturity is not significant (coefficient = 0.053, not statistically significant). This suggests that the coffee export-basket upgrading in producing countries is related solely to the absorptive capacity of their domestic markets, and that the driving role of demand maturity has not yet emerged as an independent factor. Judging from the logic of industrial evolution revealed by the Yunnan case, this outcome may be consistent with the mechanisms observed in the early stages of industrial development: domestic market absorption may initially help improve capacity utilization and build basic processing capabilities. However, due to limited purchasing power and the fact that demand for differentiated products has not yet fully developed, the maturity of demand may not yet show a significant independent correlation with the upgrading of the export basket.

The domestic market absorption effect of traditional coffee-consuming countries is no longer significant (coefficient is 0.014, not statistically significant), while the demand-maturity effect is also not statistically significant in the latest estimation (coefficient = 0.046), which aligns with the reality that per capita coffee consumption in developed markets is approaching saturation, the benefits of scale expansion are waning, and value creation is shifting toward a path-dependent system shaped by historical processing, branding, and distribution capabilities. For these mature consuming economies, the maintenance of higher export-basket sophistication depends less on further market expansion or short-term changes in demand structure than on already accumulated industrial capabilities and established positions in processing, branding, and international distribution networks. The strongly significant coefficient of the lagged dependent variable in this group further indicates that coffee export-basket upgrading in traditional consuming countries is characterized by strong historical inertia rather than being mainly driven by contemporary local market expansion. Finally, for the emerging coffee-consuming countries, the coefficients of domestic market absorption and demand maturity are significantly positive at the 10% significance level, respectively (LnDMS = 0.010; LnSTRUC = 0.037), showing a tentative “two-wheel drive” feature, indicating that the large emerging markets have the most complete dynamic mechanism to realize coffee export-basket upgrading. This statistical finding aligns with the core mechanism observed in the Yunnan case: the vast domestic market serves as a “buffer pool” for cost-sharing in the introduction of high-end processing equipment and technological upgrades, while the continuously evolving consumption structure sends a clear signal to enterprises to innovate; together, these factors drive the expansion of export products toward the high end of the value chain. Overall, the heterogeneity results show that the local market effect is strongest where domestic market absorption and demand maturity coexist. Coffee-producing countries mainly benefit from the scale effect, traditional consuming countries display strong path dependence, and emerging coffee-consuming countries are the group in which domestic demand most clearly translates into product-side upgrading in the global coffee value chain.

## 5. Conclusions, Policy Implications, and Limitations

### 5.1. Conclusions

This study examines how domestic market demand drives the upgrading of the coffee export-basket, drawing on a longitudinal case study of Yunnan’s coffee industry and panel data from 96 countries covering the period 2012–2024. The Chinese case demonstrates that industrial upgrading is not achieved directly through the expansion of market size, but rather involves a process that begins with the aggregation of resources driven by external demand, progresses through the restructuring of resources and the frictions associated with transformation that arise once differentiated demand emerges, and culminates in supply chain and network synergy driven by the mutual interplay of domestic market expansion and maturing demand. Consequently, market absorption is initially reflected in the expansion of production and basic capabilities; only when the demand structure can consistently yield returns based on quality and differentiation can these capabilities be transformed into export products that are more highly processed and of greater complexity.

The case study from Yunnan, China, provides a micro-level mechanistic explanation for the relationships proposed in H1–H3, while the cross-national analysis examines whether the corresponding macro-level relationships, nonlinear characteristics, and cross-national differences can be observed at the country-year level. The results of the GMM analysis indicate that, overall, both the absorptive capacity and the maturity of demand in the domestic market contribute to increasing the technical complexity of the coffee export basket. Threshold estimates indicate that the positive average effect absorbed by the domestic market masks the transition constraints associated with the intermediate stage of demand maturity, and its impact follows a “promotion—inhibition—re-promotion” pattern as demand maturity increases: the early stage of maturity is primarily characterized by production expansion and the accumulation of foundational capabilities; the intermediate stage is constrained by existing low-end structures and the costs of resource restructuring; and the later stage of maturity once again provides the conditions for transforming market demand into product and processing upgrades. Heterogeneity analysis further reveals that coffee-producing countries primarily benefit from domestic market absorption effects; traditional coffee-consuming countries exhibit strong path dependence rather than significant contemporaneous domestic market absorption effects; while emerging coffee-consuming countries are driven by both domestic market absorption effects and the maturity of demand. Overall, the two types of evidence are complementary: the Yunnan case illustrates how domestic demand may drive export upgrading, while the cross-country analysis identifies the general patterns of this relationship and its boundary conditions.

### 5.2. Policy Implications

Based on the research findings, this study offers the following policy recommendations: First, transform the domestic market’s absorption of domestic output into processing capacity and the upgrading of export products. Coffee-producing countries should expand domestic market access for their local output, improve processing facilities, financing conditions, and technical services, increase the utilization rate of roasting, brewing, quality testing, and packaging equipment, and stabilize the supply of raw materials through producer organizations and coordinated procurement. Emerging coffee-consuming countries should combine the expansion of their consumer markets with domestic processing, brand building, and the development of sales channels to reduce their reliance on imported processed products for consumption growth. Market variables in traditional coffee-consuming countries during the same period did not show a significant impact; therefore, policy efforts should focus on technological upgrades, product innovation, and sustainability initiatives related to existing processing, branding, and distribution capabilities. Second, foster a demand structure that can consistently yield returns on investments in quality. Quality standards, certification, and traceability systems covering grade, origin, processing methods, and sustainability should be improved. Efforts to develop geographical indications and educate consumers should be advanced, so that attributes such as flavor, origin, brand, and sustainability can lead to stable market recognition and willingness to pay. The supply side should develop differentiated products based on the resource endowments of each production region: regions with comparative advantages can focus on specialty coffee and high-quality raw materials, while large-scale production regions can expand into value-added products such as instant coffee, cold brew, and freeze-dried coffee. Additionally, they should strengthen coordination across the supply chain through producer organizations, procurement standards, and technology promotion. Third, improve support mechanisms to address transformation constraints at different stages of demand maturity. When demand maturity is low, priority should be given to improving local sales and basic processing conditions; once demand maturity is in the intermediate range, the costs of resource restructuring should be reduced through support for equipment upgrades, technical training, standards certification, and bridge financing; when demand has reached a higher level of maturity, policy resources should be directed more toward value-added processing, branding, product innovation, and the expansion of international distribution channels. At the same time, refine mechanisms such as quality premiums, long-term procurement contracts, price insurance, and regional trading platforms to improve producers’ income expectations and provide stable conditions for the sustained operation of processing capacity and the upgrading of the export product structure.

### 5.3. Limitations

This study has the following limitations that should be addressed in future research. First, the quantitative analysis in this study is based on the country-level panel data, which cannot fully capture the within-country regional variation and micro-enterprise heterogeneity. In the future, research could incorporate firm-level data or household survey data to further refine the analysis. Second, the “resource aggregation—resource orchestration—collaborative upgrading” pathway identified in the Yunnan case emerged within the specific context of the expansion of coffee consumption in China, the development of digital sales channels, and local industrial policies; different countries may follow distinct implementation pathways in terms of participating actors, resource endowments, and modes of industrial organization. Nevertheless, the case of Yunnan, China, provides evidence at the process level, revealing how domestic demand is transformed into upgraded export products through the reallocation of resources and supply chain coordination among industry participants. The indicators used in panel analysis are designed to reflect the corresponding aggregate dimensions of these relationships at the country-year level. However, the aggregated nature of panel data prevents the analysis from directly observing the processes at the participant level involved in resource reallocation and supply chain coordination. Therefore, the consistency between the findings of the case study and those of the panel data analysis should be viewed as complementary cross-level evidence: the case studies explain how the mechanism operates, while the panel data analysis assesses whether the corresponding overall relationship is observable across countries and over time. Future research could conduct comparative case studies in major coffee-producing countries such as Vietnam and Ethiopia to further identify the common elements, divergent pathways, and conditions for the application of this demand transmission mechanism across different institutional and industrial organizational contexts. Finally, this study employs a static panel threshold model [39] for testing threshold effects, rather than a dynamic panel threshold model [45,46]. Because the static threshold specification does not jointly estimate the lagged dependent variable and nonlinear regime changes, the threshold results are interpreted as complementary evidence of conditional regime differences rather than as dynamic causal effects. Future research could apply a dynamic panel threshold estimator when longer panels and stronger identification conditions become available.

## Figures and Tables

**Table 1:** Descriptive statistics table for key variables.

Variables	Definition	Mean	Std.Dev.	Min	Max
LnEXPY	Coffee export-basket sophistication	1.022	0.039	0.943	1.078
LnDMS	Domestic market absorption	0.036	0.229	−0.512	1.048
LnSTRUC	Demand maturity, proxied by real GDP per capita	0.987	0.104	0.690	1.183
Resource	Share of total natural resource rent in GP	0.043	0.064	0.000	0.485
Inflow	Share of foreign direct investment in GDP	0.059	0.345	−3.916	4.522
Manu	Manufacturing share in GDP	0.132	0.056	0.014	0.371
Gross	Share of capital stock in GDP	0.229	0.057	0.012	0.532
Lnofficial	Annual average of the official exchange rate of the local currency against the US dollar	2.927	2.999	−0.636	11.402

**Table 2:** Benchmark regression results.

	LnEXPY		
	(1)	(2)	(3)
L.LnEXPY	0.681 *** (0.097)	0.635 *** (0.094)	0.592 *** (0.099)
LnDMS	0.023 *** (0.008)	0.020 *** (0.006)	0.024 *** (0.006)
LnSTRUC		0.087 *** (0.027)	0.082 *** (0.026)
Resource			−0.044 ** (0.019)
Inflow			−0.003 * (0.002)
Manu			−0.008 (0.014)
Gross			0.006 (0.014)
Lnofficial			−0.000 (0.000)
Observations	1152	1152	1152
Fixed year effect	Yes	Yes	Yes
National fixed effect	Yes	Yes	Yes
Instrument-to-group ratio	0.156	0.167	0.219
AR (1) *p*-value	0.000	0.000	0.000
AR (2) *p*-value	0.790	0.831	0.624
Hansen *p*-value	0.208	0.807	0.850

Note: Standard errors are reported in parentheses. *, **, and *** indicated significance at the 10%, 5%, and 1% levels, respectively.

**Table 3:** Results of the threshold effect test.

Threshold Variable	Threshold Test	Initial Assumption	F-Value	*p*-Value	Critical Value		
					1%	5%	10%
LnSTRUC	Single threshold	H0: There is no threshold value	64.24 ***	0.0040	54.7496	36.0108	29.3738
	Double threshold	H0: There is a threshold value	46.90 **	0.0240	51.9144	38.2427	32.6518
	Three thresholds	H0: There are two threshold values	10.34	0.8160	60.5869	47.3711	41.2930

** and *** indicated significance at the 5%, and 1% levels, respectively.

**Table 4:** Results of the panel threshold regression.

Threshold Variable	(1)
	LnSTRUC
Resource	−0.038 ** (0.015)
Inflow	−0.003 *** (0.001)
Manu	−0.050 ** (0.027)
Gross	0.024 * (0.012)
Lnofficial	0.011 *** (0.001)
$\alpha_{1}$	0.046 *** (0.007)
$\alpha_{2}$	−0.029 *** (0.008)
$\alpha_{3}$	0.030 *** (0.008)
Observations	1248
R-squared	0.163

Note: $\alpha_{1}$ refers to domestic market absorption coefficient corresponding to the range below the first threshold; $\alpha_{2}$ refers to domestic market absorption coefficient corresponding to the range above the first threshold or between the first and second thresholds; $\alpha_{3}$ refers to domestic market absorption coefficient corresponding to the range above the second threshold or between the second and third thresholds. *, **, and *** indicated significance at the 10%, 5%, and 1% levels, respectively.

**Table 5:** Table of robustness test results.

	LnEXPY							Upgrade	
	(1)	(2)	(3)	(4)	(5)	(6)	(7)	(8)	(9)
L.LnEXPY/L.Upgrade	0.668 *** (0.108)	0.615 *** (0.103)	0.565 *** (0.109)	0.253 ** (0.103)	0.487 *** (0.148)	0.384 *** (0.142)	0.380 *** (0.100)	0.511 *** (0.126)	0.581 *** (0.115)
LnDMS	0.046 (0.029)	0.055 * (0.028)	0.053 * (0.027)	0.039 *** (0.007)	0.039 *** (0.011)	0.002 ** (0.001)	0.043 ** (0.019)	0.317 *** (0.083)	0.277 *** (0.074)
LnSTRUC		0.081 ** (0.033)	0.072 ** (0.034)	0.154 *** (0.031)	0.114 *** (0.039)	0.128 *** (0.039)	0.206 *** (0.049)	0.939 *** (0.342)	0.809 *** (0.293)
Resource			−0.063 ** (0.029)	−0.081 ** (0.024)	−0.067 ** (0.026)	−0.030 (0.021)	−0.239 *** (0.060)	−0.367 (0.259)	−0.302 (0.254)
Inflow			−0.003 ** (0.001)	−0.005 * (0.003)	−0.005 ** (0.003)	−0.004 ** (0.002)	−0.006 ** (0.003)	−0.019 *** (0.006)	−0.029 ** (0.012)
Manu			−0.000 (0.023)	−0.008 (0.019)	0.023 (0.020)	−0.215 (0.020)	−0.187 *** (0.056)	0.302 (0.194)	0.196 (0.180)
Gross			0.003 (0.020)	0.003 (0.018)	0.009 (0.016)	0.016 (0.022)	0.043 (0.029)	0.021 (0.157)	−0.021 (0.147)
Lnofficial			−0.001 (0.001)	−0.001 (0.001)	−0.000 (0.001)	−0.000 (0.001)	−0.002 (0.001)	−0.002 (0.006)	−0.003 (0.005)
Observations	1152	1152	1152	960	1152	1152	1152	1152	1152
Fixed year effect	Yes	Yes	Yes	Yes	Yes	Yes	Yes	Yes	Yes
National fixed effect	Yes	Yes	Yes	Yes	Yes	Yes	Yes	Yes	Yes
Instrument-to-group ratio	0.167	0.177	0.229	0.198	0.219	0.219	0.219	0.219	0.219
AR (1) *p*-value	0.000	0.000	0.000	0.008	0.000	0.013	0.000	0.000	0.000
AR (2) *p*-value	0.793	0.846	0.666	0.969	0.488	0.931	0.709	0.956	0.543
Hansen *p*-value	0.351	0.697	0.743	0.203	0.496	0.411	0.689	0.921	0.945

*, **, and *** indicated significance at the 10%, 5%, and 1% levels, respectively.

**Table 6:** Table of heterogeneity analysis results.

	LnEXPY		
	(1) Coffee-Producing Countries	(2) Traditional Coffee-Consuming Countries	(3) Emerging Coffee-Consuming Countries
L.LnEXPY	0.487 *** (0.164)	0.869 *** (0.062)	0.720 *** (0.104)
LnDMS	0.033 *** (0.008)	0.014 (0.012)	0.010 * (0.005)
LnSTRUC	0.053 (0.038)	0.046 (0.031)	0.037 * (0.020)
Resource	−0.007 (0.029)	−0.064 (0.049)	−0.026 * (0.014)
Inflow	0.083 (0.050)	−0.003 *** (0.001)	−0.002 (0.004)
Manu	−0.012 (0.049)	−0.014 (0.013)	−0.022 (0.015)
Gross	0.046 (0.028)	0.007 (0.020)	−0.011 (0.011)
Lnofficial	−0.001 (0.001)	−0.001 (0.001)	−0.000 (0.000)
Observations	324	288	540
Number of countries	27	24	45
Fixed year effect	Yes	Yes	Yes
National fixed effect	Yes	Yes	Yes
Instrument-to-group ratio	0.78	0.88	0.47
AR (1) *p*-value	0.009	0.089	0.003
AR (2) *p*-value	0.718	0.569	0.635
Hansen *p*-value	0.739	0.917	0.654

* and *** indicated significance at the 10% and 1% levels, respectively.

## Data Availability

The country-level data used in this study were obtained from the following databases: UN Comtrade Plus (http://comtradeplus.un.org/) and the CEPII BACI database (https://www.cepii.fr/CEPII/en/bdd_modele/bdd_modele_item.asp?id=37)for international trade data; the USDA Production, Supply and Distribution database (https://apps.fas.usda.gov/psdonline/app/index.html#/app/advQuery) and FAOSTAT (https://www.fao.org/faostat/en/#data) for coffee production, supply, and consumption data; the World Bank World Development Indicators database (https://databank.worldbank.org/source/world-development-indicators) for macroeconomic indicators; and the Worldwide Governance Indicators database (https://databank.worldbank.org/source/worldwide-governance-indicators) for governance-related indicators. Alternative coffee-market expenditure data were obtained from Statista (https://www.statista.com/). The interview data are available from the corresponding author upon reasonable request and are not publicly available to protect participant confidentiality.

## Supplementary Materials

The following supporting information can be downloaded at: https://www.mdpi.com/article/10.3390/foods15183240/s1, Table S1. Typical evidence citation examples at the Quality-oriented transformation stage. Table S2. Typical evidence citation examples at the Brand-building transformation stage. Table S3. Key Interview Subjects and Topics in Yunnan’s Coffee Industry. Table S4. Basic Characteristics of Coffee Companies (N = 223). Table S5. Basic Characteristics of Coffee Farmers (N = 300). Table S6. Basic Characteristics of Key Respondents from Government Agencies (N = 139). Table S7. Basic Characteristics of Consumers (N = 350). Table S8. Information on secondhand materials.

## Appendix A

**Table A1:** Typical evidence citation examples at the raw material source stage.

Aggregate Dimension	Second-Order Category	First-Order Concept	Illustrative Examples
Resource Situation	External Demand Leading	Reliance on bulk exports	In the early days, over 90% of our green coffee beans were exported directly in burlap sacks, primarily to fill the supply gap for bulk coffee beans in Europe and the United States (O11).
		Captured by multinational giants	Foreign-invested companies held absolute sway, setting purchase prices by deducting several dozen cents from New York futures prices, which drastically squeezed the net profit per mu that coffee farmers earned after a year of hard work (E21); the Catimor variety is easy to grow… yet coffee farmers’ annual income was only 10,000 yuan (EX3).
	Weaknesses In Internal Capabilities	Front-end production is rudimentary	At that time, the primary focus was on yield; farmers planted whatever was easy to grow, field management was rudimentary, and there was no scientific guidance on agricultural practices (A11); pest and disease problems were severe… and there was limited interaction with outside services (S13).
		Gap in advanced processing technology	There is virtually no modern high-temperature roasting equipment in the country, resulting in very low yield rates for shelling and primary processing, and persistently high rates of defective products (A31).
		Back-end capabilities are weak	We have no control over the market… a reduction of a few yuan (A32); foreign investors, seeking to maximize their profits… farmers may end up with less than 0.5 yuan (EX2).
Resource Bricolage	Polycyclic Chain Filling	Targeting industry leaders	Selectively focus on areas with a strong foundation… to increase market share (A11); Learning is a never-ending process… persist in learning from the industry’s best… a key factor in success (EX1).
		Build a basic full-industry chain	Advanced processing and quality… require strict quality control (A11); optimizing advanced processing… to foster industrial clustering (S13).
	Factor Empowerment	Leveraging local advantages	Yunnan’s greatest asset is its land located within the “golden latitude belt” (EX1); its unique geographical and climatic conditions provide the ideal environment for producing high-quality coffee (EX2); and compared to other regions around the world, it boasts an abundant and inexpensive labor force… with more than 1 million coffee farmers (IN1).
		Acquiring external resources	Four varieties of high-quality coffee seedlings were imported from Costa Rica for experimental cultivation (EX2); the new factory has introduced German coffee roasting equipment as well as advanced foreign equipment and technology… to establish a customized production model (EX1).
Value Chain Creation	Upgrade of OEM Chains	Industrial clustering started	“China’s coffee market has seen a compound annual growth rate exceeding 25%, driving the shift from selling raw materials to selling finished products” (EX1); Hougu Coffee focuses on developing the province’s walnut, coffee, and tea industries… offering better taste and greater health benefits (E11); these three industries are fostering local coffee brands in Yunnan (E22).
		Integration of agriculture, industry, and trade	Training for the “Demonstration and Promotion of an Integrated Development Model for the Industrial Chain of Specialty Tropical Crops” Project (EX3).
	Obtain the Base Rent	Ricardian rent	China’s export logistics chain continues to operate smoothly and steadily… delivering impressive results through trade transactions (EX1); China’s coffee industry… expanding international trade and tapping into the domestic market’s potential (EX3).
		Establishing economies of scale	The total agricultural output value of the province’s coffee industry reached 3.6 billion yuan… Thirty high-standard primary processing plants were constructed (EX3); several large-scale primary processing and distribution centers have gradually emerged in Pu’er and Baoshan, marking a transition from small-scale, individual farming to large-scale cultivation (EX1).

Note: All interviews were conducted in Chinese and transcribed verbatim. To ensure the accuracy of the English quotations, we followed cross-cultural research guidelines and implemented a standard back-translation procedure. First, a researcher who is well-versed in this field of study and is bilingual translates the original Chinese text into English. Subsequently, another independent bilingual researcher (who had not seen the original written record) back-translated the English text into Chinese. The research team discussed and resolved the discrepancies between the original Chinese version and the back-translated Chinese version to ensure semantic equivalence. All content cited in this article has been cross-checked against the original transcripts to ensure complete accuracy.

**Table A2:** Table of correlation coefficients.

	LnEXPY	LnDMS	LnSTRUC	Resource	Inflow	Manu	Gross	Lnofficial
LnEXPY	1.000							
LnDMS	0.349 ***	1.000						
LnSTRUC	0.700 ***	0.137 ***	1.000					
Resource	−0.331 ***	0.160 ***	−0.323 ***	1.000				
Inflow	−0.002	−0.040	0.074 ***	−0.072 **	1.000			
Manu	0.094 ***	−0.059 **	0.171 ***	−0.210 ***	−0.087 ***	1.000		
Gross	−0.092 ***	0.056 **	−0.099 ***	0.132 ***	−0.045	0.216 ***	1.000	
Lnofficial	−0.441 ***	0.023	−0.580 ***	0.346 ***	−0.081 ***	0.053 *	0.213 ***	1.000

*, **, and *** indicated significance at the 10%, 5%, and 1% levels, respectively.

**Table A3:** Multicollinearity test table.

Variable	VIF	1/VIF
LnDMS	1.09	0.916998
LnSTRUC	1.75	0.57244
Resource	1.31	0.765439
Inflow	1.03	0.968393
Manu	1.21	0.827958
Gross	1.12	0.893284
Lnofficial	1.73	0.577479
Mean VIF	1.32	-

**Table A4:** National classification table.

Category	Countries
Coffee-producing countries	Angola; Burundi; Bolivia; Côte d’Ivoire; Cameroon; Costa Rica; Dominican Republic; Ecuador; Ethiopia; Ghana; Guinea; Guatemala; Honduras; Jamaica; Kenya; Lao People’s Democratic Republic; Madagascar; Malawi; Nigeria; Nicaragua; Panama; Peru; Papua New Guinea; Sierra Leone; El Salvador; United Republic of Tanzania; Uganda
Traditional coffee-consuming countries	Australia; Austria; Belgium; Canada; Switzerland; Cyprus; Germany; Denmark; Spain; Finland; France; United Kingdom of Great Britain and Northern Ireland; Greece; Ireland; Italy; Japan; Luxembourg; Malta; Netherlands; Norway; New Zealand; Portugal; Sweden; United States of America
Emerging coffee-consuming countries	Albania; Argentina; Armenia; Bulgaria; Bosnia and Herzegovina; Brazil; Chile; China; Colombia; Czechia; Algeria; Egypt; Estonia; Georgia; Hungary; Indonesia; India; Iran (Islamic Republic of); Jordan; Kazakhstan; Republic of Korea; Lebanon; Lithuania; Latvia; Morocco; Mexico; North Macedonia; Montenegro; Malaysia; Philippines; Poland; Romania; Russian Federation; Saudi Arabia; Senegal; Singapore; Serbia; Slovakia; Slovenia; Thailand; Turkey; Ukraine; Uruguay; Viet Nam; South Africa
