# Supplementary material for: How Domestic Demand Drives Coffee Export-Basket Upgrading: Pathways to Agri-Food System Transformation
Source: Foods. 2026 Sep 14;15(18):3240. doi: 10.3390/foods15183240 (PMC13605605; doi:10.3390/foods15183240)
Supplement: Supplementary file 1 [file foods-15-03240-s001.zip › foods-4495480-supplementary.pdf]

Supplementary Materials

Table S1. Typical evidence citation examples at the Quality-oriented transformation stage.

Aggregate Dimension	Second-Order Category	First-Order Concept	Illustrative Examples
Resource Situation	Niche-Induced	A preference for high-value-added emerged	Coffee consumers... the vast majority of whom are no longer driven solely by functional needs... are beginning to shift toward psychological and social needs (OB2); consumption upgrades and generational shifts have significantly fueled a growing appreciation for quality (EX1).
		Traceability and identification of regional flavors	Yunnan is characterized by its low latitude and high altitude… With approximately 300,000 mu of Starbucks Certified Plantations (EX2), Yunnan coffee relies on its unique highland climate… making it a must-have ingredient for many internationally renowned companies (EX3).
	Transition Constraints	High upfront costs	Fresh fruit picking requires 24-hour processing... resulting in high labor costs (G12); a key industry challenge is the small and fragmented scale of operations... leading to insufficient value extension (E22); and upgrading washing and fermentation equipment places an extremely heavy burden on small and medium-sized farmers (G33).
		Midstream financial constraints	Although various departments at all levels have introduced measures to varying degrees... overall funding remains scarce and fragmented... and support is insufficient (E21); while everyone is eager to produce high-quality beans, introducing advanced digital temperature-controlled roasting production lines requires substantial capital, and financing options are extremely limited (E22).
		Weak downstream power	The current state of the coffee industry... does not hold a competitive advantage at the consumer end... capital operation models (G11); the coffee supply market is mature... with frequent price fluctuations (G12); the development of coffee-related industries lags behind... and has not yet been fully tapped (G33).
Resource Orchestration	Strengthening the Vertical Supply Chain	Establish a comprehensive quality control system	Increase the proportion of premium and high-quality beans in primary production... Ensure that fresh cherry processing meets standards... Enhance wastewater treatment capacity (S13); Establish a comprehensive standards system from primary processing to sales... Promote high-quality development of Yunnan's coffee agriculture through standardization (A31); We have begun signing minimum-price purchase agreements with farmers, implementing strict harvesting standards, and securing a supply of high-quality fresh cherries by providing organic fertilizer and technical guidance (S13).
		Innovative models of industrial integration	We must move beyond a business model focused solely on agriculture (P11); Baoshan is accelerating the integrated development of the "coffee + tourism" industry... innovating new models of coffee consumption... and creating a new trend and calling card for Baoshan tourism (EX1).

Aggregate Dimension	Second-Order Category	First-Order Concept	Illustrative Examples
Chain Cluster Value Creation	Empowered by Core Technologies	Application of introduced superior germplasm	Continuously introduce germplasm... to establish a stable extension model involving "research institutes, enterprises, and farmers" (E38); provide substantial fiscal support for infrastructure... and distribute subsidies for research, labor, and extension services (S13).
		Focusing on advanced processing techniques	Value-added processing is a core component of the industry… The plan is to reach 100,000 and 200,000 metric tons within five years (A32); we will strive to enhance our value-added processing capabilities and strictly control processing procedures (A11).
	Product and Business Model Evolution	Differentiated flavors	The production of Arabica coffee beans in Yunnan has been growing steadily… Their quality is on par with that of Central American coffee beans (EX3); rising prices for fresh coffee cherries… have provided strong momentum for the sustainable and healthy development of the coffee industry (EX2); through the development of specialized processing methods such as anaerobic fermentation and sun-drying, the cupping scores of Yunnan's specialty beans have consistently remained above 80 points, making them fully capable of replacing imported beans (EX1).
		Business model innovation	New internet-based coffee shops and Luckin's online ordering with offline delivery (EX8). In the primary sector, strive to achieve estate-style development as much as possible… as an innovative integration model (E38).
	Market Forces Reshaping the Market	Chamberlinian rent	Bringing high-quality beans directly to consumers and breaking down existing industry barriers... has created a win-win situation for both new and veteran coffee farmers (EX1); "Yunnan coffee is gradually becoming synonymous with high-quality coffee" (EX3).
		Competing for international pricing power	Pu'er City is currently working to establish local coffee brands... to regain control over international pricing (EX1); and to strengthen cooperation with major online platforms... to build a regional brand moat (S13).

Table S2. Typical evidence citation examples at the Brand-building transformation stage.

Aggregate Dimension	Second-Order Category	First-Order Concept	Illustrated Examples
Resource Situation	Driven by Demand Growth	Domestic demand surges	In 2022, national coffee consumption reached 229,000 metric tons, a 25.37% increase from the previous year. Coffee consumption is growing at a remarkable annual rate of over 15% (IN1); the explosive growth in domestic demand has significantly stimulated prosperity in the downstream segment of the industry chain (S11).
		Cutting-edge strategies for cross-industry brands	A host of domestic brands such as Manner, Seesaw, and 3TonBan have emerged (EX9); Li-Ning, China Post, Tong Ren Tang, Sinopec, and Huawei have all expanded into the coffee industry (EX10).
	Overcoming the Barriers to Transformation	Front-end quality lock price breakout	China's potential for expanding coffee cultivation is already quite limited… The rate of high-quality coffee stands at 10%, and while the rate of specialty coffee is only 5%, it is rising rapidly

Aggregate Dimension	Second-Order Category	First-Order Concept	Illustrated Examples
			(E22); under government guidance, coffee farmers are gradually overcoming challenges in their management practices (S13).
		The weak board in the middle and back end has been fully made up	Local factories have already mastered world-leading technologies for the extraction of freeze-dried powders and cold-brewed liquids, enabling flexible, customized production to keep pace with the rapid product iterations of leading new retail brands (G33); some companies are quietly transitioning toward value-added processing… enhancing product value through independent R&D (EX11); and shifting from a lack of brand awareness to significant investments in brand building, thereby breaking free from the constraints of short-term gains (A11).
	Network-Based Chain Extension Restructuring	Horizontal and vertical chain strengthening	Develop coffee-related industries, independently research and develop coffee grinders and coffee processing equipment... and expand the value chain" (G11); "Develop coffee plantations, offer coffee education, and fully promote the integration of the primary, secondary, and tertiary sectors (G12).
Resource Scheduling		Policy guidance	The Yunnan Provincial Department of Agriculture and Rural Affairs has issued the "Measures for the Certification of Premium Coffee Estates" to accelerate the improvement of quality and efficiency as well as the transformation and upgrading of the province's coffee industry (IN2).
	Holistic Ecological Empowerment	Building a public brand matrix	Vigorously build a comprehensive public brand... that showcases the distinctive characteristics of each production region while preserving the individuality of smaller brands (E22); provide policy support through a unified service platform and promote the brand through a unified coffee culture festival (G41).
		Integrated platform services	Policy support for the unified service platform and coordinated promotion of the Coffee Culture Festival (G41); as a third-party service platform, our team will work diligently to advance the industry… striving to achieve the goals of enriching the people and strengthening the city (E34).
		Diverse innovation in high-value-added products	In addition to three-in-one products, there are other products that can be developed. For example, the utilization of coffee by-products and the reuse of pectin, fruit, flower stalks, pulp, and peel (A11); our city has developed new coffee varieties, "Yunka No. 1" and "Yunka No. 2" ... integrating into the global market (EX1).
Chained-Network Style Value Co-Creation	Chained-Network Upgrading	New business models combining coffee and travel	Simao District in Pu'er City, Yunnan Province, has forged a path to prosperity for farmers by integrating coffee production with tourism. In 2021, the per capita disposable income of coffee farmers in Simao District reached 12,000 yuan (EX13); in 2021, the output value of Yunnan's entire coffee industry chain was 31.672 billion yuan, representing a year-on-year increase of 1.72%. Agricultural output reached 2.643 billion yuan, processing output reached 17.362 billion yuan, and wholesale and retail value-added reached 11.667 billion yuan, all setting new historical highs and demonstrating significant results (EX3).
	Value Co-Creation	Schumpeterian rents	By promoting new technologies such as community-based cultivation and green pest and disease control... Yunnan's coffee cultivation and deep-processing technologies have achieved international leadership (EX3); its leading industrial clusters

Aggregate Dimension	Second-Order Category	First-Order Concept	Illustrated Examples
		An inclusive society and shared prosperity	have been included in the Ministry of Agriculture and Rural Affairs' 2022 development list (EX14); we have not only achieved environmentally friendly and sustainable cultivation but, through a robust domestic market... have also driven the local coffee industry's deep integration into international quality evaluation systems, laying a solid foundation for substantially influencing global coffee product standards in the future (EX5). More and more coffee farmers are enjoying a better quality of life, and in the future, we hope that through the efforts of our younger generation, even more people will have the opportunity to taste Yunnan coffee (EX15); an increasing number of young people are choosing to return to their hometowns to start businesses, using the knowledge and skills they've acquired to help local coffee farmers overcome the dilemma where the income from one kilogram of coffee beans isn't enough to buy even a single cup of coffee (EX16).

Table S3. Key Interview Subjects and Topics in Yunnan's Coffee Industry.

Name of the Interviewed Organization		Interview Transcript	Key Participants (number)	Duration (approx.)
Relevant Government Agencies	"Green Food Brand" Division, Yunnan Provincial Department of Agriculture and Rural Affairs	The development history, current status, and challenges of Yunnan's coffee industry; the effectiveness of policies and plans for the industry's development; regional endowments, product quality, base construction, and policy-based coffee insurance at the front end; value-added processing at the mid-stage; regional public brands, cultural promotion, market development, development positioning, and development goals at the back end; coffee competitions and events; renowned local enterprises and brands; market expansion through new consumption models; integrated industrial development; technological support; and the development of supporting infrastructure systems.	3	120 minutes
	Pu'er Municipal Bureau of Agriculture and Rural Affairs, Center for Coffee and Tea Industry Development	The status of coffee production and operations in the region; coffee germplasm breeding, cultivation, processing, storage, distribution, sales, and imports and exports; the operational status of existing production bases; coffee estates; coffee brand development; and industrial support policies and their implementation.	8	100 minutes
	Dehong Prefecture Bureau of Agriculture and Rural Affairs		6	120 minutes
	Baoshan City Bureau of Agriculture		2	90 minutes

Name of the Interviewed Organization		Interview Transcript	Key Participants (number)	Duration (approx.)
Front-End Main Content	Ning'er County Government	Coffee industry resources, cultivated varieties, scale of cultivation, farmers’ yields, target-price insurance, subsidy policies, agricultural technical services, investment promotion and cooperation in value-added processing, integrated industrial development, major challenges and issues facing local coffee farmers and enterprises, supporting policies and measures for the coffee industry, as well as the planning and implementation of the coffee industry.	9	90 minutes
	Mangshi Bureau of Agriculture and Rural Affairs		2	90 minutes
	Lujiang Town Government		3	60 minutes
	Baoshan City Coffee Industry Development Task Force	Coffee cultivation area and industry planning; variety renewal, understory cultivation, and mechanized harvesting; environmentally friendly processing of fresh coffee cherries, value-added processing, and product development; integration of culture and tourism, inclusive policies, financial support, and collaboration among brands and growing regions; constraints related to land, financing, talent, and infrastructure; production technologies and enterprise evaluation standards; regional differentiation, brand building, coffee tree adoption programs, and specialty coffee evaluation systems.	3	130 minutes
	Coffee Plantation in Dakahe Village, Simao District	General information on the operation of coffee production bases; the current status of coffee development, including the purchase of seedlings, planting, cultivation, maintenance, harvesting, primary processing, and sales; and the integration of coffee and tourism.	8	180 minutes
	Cai Zhi Coffee Cooperative, Wangjie Village, Liming Township, Ning'er County	Operational aspects of different management models for coffee cultivation and sales—including “company + farmer,” “company + plantation + farmer,” and “company + plantation + cooperative”—cover land use, cultivation, primary processing, organized procurement, the supply of production inputs to members, sales, and technical guidance.	5	120 minutes
	Jiangxi Village Riverside Farmers' Planting Cooperative, Mohei Town, Ning'er County		6	120 minutes
	Mangshi Soil and Fertilizer Station	In areas such as the breeding of high-quality coffee varieties, the optimization of cultivation practices, and the provision of soil data needed by downstream sectors.	4	60 minutes
	“One Village, One Product” Model Village—Xinzhai Village, China's Number One Coffee	Introduction of plant varieties, cultivation, primary processing, sales, and the provision of technical training, technical exchanges, and consulting services related to cultivation and management.	3	60 minutes

	Name of the Interviewed Organization	Interview Transcript	Key Participants (number)	Duration (approx.)
	Village			
	Yunnan Hongji Coffee Industry Co., Ltd.	Coffee cultivation, primary coffee processing, and import/export trade	1	100 minutes
	Baoshan Chunzhen Coffee Industry Development Co., Ltd.	The "company + base + specialized cooperative + farmers + research institutions" development model; the "farmers + company + customers" contract farming model; and the establishment of an agricultural product traceability system.	1	100 minutes
	Laos Changshengda Coffee Company	The coffee brand "Lao Coffee," poppy substitution projects, eco-friendly specialty coffee cultivation, coffee plantations, primary and value-added coffee processing, and partnerships with well-known brands.	6	150 minutes
Mid-range model	Yunnan Hougu Coffee Co., Ltd.	The state of the coffee processing industry, including procurement, processing, sales, trade agency services, technological innovation in processing methods, production line development, agricultural technology extension services, product category development, processing licensing, quality inspection and environmental protection, financial subsidies, industrial park development, operational center construction, industrial chain collaboration, the construction of integrated warehousing bases linked to comprehensive bonded zones, and the production and manufacturing of coffee derivatives.	1	140 minutes
	Yunnan Zhongka Coffee Co., Ltd.		3	90 minutes
	Baoshan Jinqing Tropical Crops Technology Co., Ltd.		3	90 minutes
	Baoshan Small-Bean Coffee Processing Park		2	30 minutes
	Luang Prabang Saffron Coffee Roasting Company		2	120 minutes
	Yunnan Four Cats Coffee Co.,	Instant coffee market, market sales, industry competition, e-commerce operations, brand marketing, consumer education, supply chain collaboration, policy requirements.	1	120 minutes
Backend Body	Ltd.	The specialty coffee market, investments in coffee shops, coffee shop services, operations and sales, licensing, evolving consumer demand, the development of coffee's multifaceted attributes, market channel outreach to end consumers, storage facilities, delivery centers, division of labor and collaboration within the industry chain, and new domestic brands.	1	120 minutes
	The Way of Bitterness Coffee Co., Ltd.	Xiaoli Coffee's digital and brand transformation; regions where Xiaoli Coffee is primarily distributed and sold.	2	30 minutes
	Baoshan Xiaoli Coffee Logistics Park	Coffee Culture; The Story of Yunnan Small-Bean Coffee; Small-Bean Coffee Art and Merchandise.	2	30 minutes
Entities	Baoshan Xiaoli	Core business: A full-industry-chain operation model integrating	2	60 minutes

Name of the Interviewed Organization		Interview Transcript	Key Participants (number)	Duration (approx.)
Driving Industrial Convergence	Coffee Cultural District	coffee R&D, cultivation, processing, warehousing, sales, and training; logistics services; warehousing services; and agricultural technical services. An integrated industrial development model combining agriculture, industry, tourism, and culture—including coffee consumption, cultivation experiences, nature education, coffee tourism, food and beverage services, and estate hotels. This model holds strategic, brand, and economic significance for preserving local culture, revitalizing rural areas, and promoting the development of the coffee industry across the region. Brand building, product development, coffee-themed cultural tourism, integration of the primary, secondary, and tertiary sectors, one-stop services, Fair Trade certification, cooperation with multinational corporations, coffee estate development, exploration of cultural heritage, coffee product quality testing centers, supply chain collaboration, introduction of global specialty coffee quality control systems, cultural promotion, and targeted marketing.		
	Yunnan Manye Coffee Estate		3	110 minutes
	Simao Beigui Coffee Co., Ltd.		1	90 minutes
	Pu'er Changmu Coffee Co., Ltd.		1	100 minutes
	Pu'er Aini Coffee Co., Ltd.		2	60 minutes
	Nanhu Gaoligong Coffee Center		2	120 minutes
	Mountain Top No. 1 Estate		1	180 minutes
	Xinzhai Coffee LLC		3	90 minutes
	Baoshan Bitton Coffee Co., Ltd.		4	120 minutes
	Huazhici Technology Co., Ltd.		3	90 minutes
	Gaosheng Estate	Full-chain operations, collaborative farming, and green bean processing; corporate quality control, employee training, and standardized management; technical barriers such as workforce stability, support for the secondary sector, and fermentation; as well as research on regional characteristics and the functional properties of coffee.	3	90 minutes
	Xinzhai Coffee	Tepica cultivation, understory farming, and contract farming; green coffee bean trade, roasting, branding, and coffee-themed tourism; specialty estate farming, mechanization, financing, sales and marketing, and research on the medicinal and dietary uses of coffee.	2	120 minutes
Other Entities	Pu'er Fumin Agricultural Equipment Co., Ltd.	Research and development, manufacturing, and sales of agricultural and household machinery and equipment for coffee processing; quality control and environmental protection; technological innovation; patent acquisition; and overseas sales.	2	90 minutes
	Yunnan Aofu Industrial Co., Ltd.		4	90 minutes
	Jinlu Agricultural and Sideline Products Co., Ltd.	Current Status of Coffee By-Product Suppliers; Coffee Cultivation and Processing; Brand Registration for Coffee Plantations; Research on Coffee Roasting; Identification of Market Demand; Promotion of Coffee Brand Experience Stores.	2	90 minutes
	Gaolaozhuang Agricultural and		2	120 minutes

Name of the Interviewed Organization		Interview Transcript	Key Participants (number)	Duration (approx.)
	Sideline Products Co., Ltd.			
	Xishuangbanna Dongjie Trading Co., Ltd.	Trade agency services; sales of daily necessities, agricultural and sideline products, prepackaged foods, and bulk foods; import and export of goods and technology.	3	120 minutes
	Helai Coffee	Commercial coffee bean imports and exports, the European market, and the coffee trade with Myanmar; financing and settlement cycles; quality inspection, cupping, standardized production, price fluctuations, supply shortages, and alternative crops.	2	90 minutes
Research Institutes	Institute of Cash Crops, Yunnan Academy of Agricultural Sciences	Achievements in building the coffee breeding system; introduction of germplasm resources, variety selection and breeding, and patent protection; technology transfer; construction of supporting infrastructure for cultivation; investment in research on	9	310 minutes
	Dehong Tropical Agricultural Science Research Institute, Yunnan Province	value-added processing; warehousing and logistics; development of supporting services for business incubation; fiscal support; establishment of a full-industry-chain standards system; construction of testing centers; research collaboration platforms; specialty coffee cultivation, pest and disease control, mechanized harvesting, and water and fertilizer management; farmer participation; and policies for evaluating specialty coffee estates.	8	180 minutes
	China Coffee Engineering Research Center	Primary processing and value-added processing technologies, processing workflows, costs of different production steps, quality assessment methods, etc.	6	180 minutes
Relevant Supporting Entities	Yunnan Coffee Industry Association	The history of the coffee industry’s development; the association’s primary responsibilities; information on member companies; the development of a unified quality standards system; quality testing; guidance on corporate brand innovation and product development; industry cooperation and exchange; and future prospects.	1	160 minutes
	Yunnan Federation of Industry and Commerce	The status of enterprises across the upstream, midstream, and downstream segments of the coffee industry chain; the status of corporate collaboration; quality standards; and industry expansion.	1	120 minutes
	Yunnan International Coffee Trading Center	Transaction volume, number of visitors, participating merchants, coffee experiences, product exhibitions and sales, warehousing, logistics, and distribution, online and offline transactions, coffee training centers, and specialty coffee processing parks.	1	60 minutes
	Luang Prabang Chamber of Commerce	Promoting alternative crops, fostering connections among companies within the industry, facilitating communication between coffee companies and the government, coordinating	8	120 minutes

Name of the Interviewed Organization	Interview Transcript	Key Participants (number)	Duration (approx.)
	industry interests, and safeguarding the legitimate rights and interests of member companies to promote the industry's development.		
Baoshan Industrial Park	Industrial layout of the park and coffee output value; coffee industry clustering, logistics, and financing constraints; bonded warehouses, export supervision warehouses, spot trading, logistics digitization, and investment promotion collaboration.	3	70 minutes

Note: This table summarizes the interviewees and topics covered in interviews conducted with key industry stakeholders during the study period. It is primarily intended to illustrate the industrial sectors and issues addressed in the interview data.

Table S4. Basic Characteristics of Coffee Companies (N=223).

Statistical Variables	Sample Distribution		Statistical Variables	Sample Distribution	
Company Age:	Number	Percentage	Number of Employees:	Number	Percentage
10 years or less	50	22.42%	20 or fewer	36	16.14%
11–20 years	92	41.26%	21–30	71	31.84%
21–30 years	67	30.04%	31–100	76	34.08%
Over 30 years	14	6.28%	More than 100	36	16.14%
			Did not answer	4	1.79%
Asset Size:	Number	Percentage			
Less than 10,000,000	118	52.91%			
10,000,000–15,000,000	50	22.42%			
15,000,000–20,000,000	45	20.18%			
More than 20,000,000	10	4.48%			

Note: N represents the number of coffee companies included in the statistics.

Table S5. Basic Characteristics of Coffee Farmers (N=300).

Statistical Variables	Sample Distribution		Statistical Variables	Sample Distribution	
Age:	Number	Percentage	Level of Education:	Number	Percentage
20–34	18	6.00%	Elementary school or below	131	43.67%
35–45	75	25.00%	Junior high school	88	29.33%
46–56	91	30.33%	High school/Technical secondary school/Vocational school	59	19.67%
57–67	52	17.33%	Junior college/Higher vocational school	21	7.00%

67 and older	64	21.34%	Bachelor's degree	1	0.33%
			Master's degree or higher	0	0
Age of Trees:	Number	Percentage			
5 years or less	20	6.67%			
6–10 years	37	12.33%			
11–20 years	157	52.33%			
21–40 years	50	16.67%			
Over 40 years	36	12.00%			

Note: N represents the number of coffee farmers included in the statistics.

Table S6. Basic Characteristics of Key Respondents from Government Agencies (N=139).

Statistical Variables	Sample Distribution		Statistical Variables	Sample Distribution	
Age:	Number	Percentage	Level of Education:	Number	Percentage
15–25 years old	17	12.23%	Elementary school or below	0	0
26–35 years old	18	12.95%	Junior high school	0	0
36–45 years old	41	29.50%	High school/Technical secondary school/Vocational school/Vocational high school	6	4.31%
46–55 years old	55	39.57%	Junior college/Higher vocational school	40	28.78%
55 years old and older	8	5.76%	Bachelor's degree	75	53.96%
			Master's degree or higher	18	12.95%
Job Grade:	Number	Percentage	Years of Experience:		
Inspector	15	10.79%	1–5 years	60	43.16%
Research Officer	3	2.16%	6–10 years	41	29.50%
Senior Section Officer	19	13.67%	11–15 years	14	10.07%
Section Officer	65	46.76%	15+ years	24	17.27%
Senior-Level (Full)	13	9.35%			
Senior-Level (Associate)	11	7.91%			
Intermediate-Level	5	3.60%			
Assistant-Level	8	5.76%			

Note: The sample covers government departments across different regions and administrative levels, with one primary representative selected from each department; N represents the number of representatives included in the analysis.

Table S7. Basic Characteristics of Consumers (N=350).

Statistical Variables	Sample Distribution		Statistical Variables	Sample Distribution	
Gender:	Number	Percentage	Level of Education:	Number	Percentage
Male	184	52.57%	Elementary school or below	3	0.86%
Female	166	47.43%	Junior high school	4	1.14%
			High school/Technical secondary school/Vocational school/Vocational high school	24	6.86%
			Junior college/Higher vocational school	105	30.00%
			Bachelor's degree	122	34.86%
			Master's degree or higher	92	26.29%
Age:	Number	Percentage	Occupation:		
15 and under	6	1.71%	Student	109	31.14%
16–35	178	50.86%	Computer Science/Internet/Telecommunications/Electronics	38	10.86%
36–45	97	27.71%	Travel/Hospitality/Food Service	32	9.14%
46–55	62	17.72%	Advertising/Marketing/Media	30	8.57%
55 and older	7	2.00%	Accounting/Finance/Banking/Insurance	26	7.43%
			Consulting/Law/Education/Research	27	7.71%
			Sales/Customer Service/Technical Support	19	5.43%
			Human Resources/Administration/Senior Management	18	5.14%
			Construction/Real Estate	15	4.29%
			Biotechnology/Pharmaceuticals/Healthcare	9	2.57%
			Other	27	7.72%

Note: N represents the number of coffee consumers included in the survey.

Table S8. Information on secondhand materials.

**Data Sources**	**Name**	**Category**	**Quantity**

Internal Sources	Government archives, statistical yearbooks, annual reports, internal publications of the coffee industry association, and the special issue of *Pu'er Coffee*	Document	22
	Official documents, implementation plans, and planning reports issued by relevant departments at various levels, including the Yunnan Provincial Department of Agriculture and Rural Affairs, the Provincial Department of Finance, the Office of the People's Government of Dehong Prefecture, the Pu'er Municipal Bureau of Agriculture and Rural Affairs, and the Ning'er County Government	Document	48
	Yunnan Academy of Agricultural Sciences Series on Coffee Industry Development	Books	2
	Company Summaries and Management Speeches from Leading Companies in the Industry	Document	5
External Sources	News reports on the coffee industry by mainstream news outlets such as the People's Daily, Guangming Daily, China Daily Online, Toutiao, Sohu, NetEase News, Sina, and Yunnan Daily	News	608
	Materials from third-party platforms such as journal articles and WeChat official accounts	Article	1895
	Official industry and corporate sources, including the official website of the International Coffee Organization, Coffee Finance Network, the official website of the Department of Agriculture and Rural Affairs, and the WeChat official accounts and websites of leading enterprises	Article	468
	Series on coffee industry giants such as *The World of Coffee* and *Starbucks*	Books	4
	Coffee Industry Market Report	Document	95
	The film *Just a Short Drive Home*, the Yunnan coffee promotional videos *Glory and Dreams* and *The Coffee Factory*, the documentary *A Call from Deep Within the Colorful Clouds*, and promotional videos for leading enterprises	Videos	10
Non-participatory observation	Participated in industry training sessions and industry development symposiums, including the Advanced Training Course on High-Quality Development of the Coffee Industry organized by the Yunnan Academy of Agricultural Sciences	Field Observations	5
	Visit coffee plantations, primary processing facilities, secondary processing facilities, coffee trading centers, specialty coffee shops, e-commerce live-streaming sales, coffee estates, coffee exhibitions, coffee culture festivals, and more	Field Observations	16
